# A Targeted Mass Spectrometric Analysis Reveals the Presence of a Reduced but Dynamic Sphingolipid Metabolic Pathway in an Ancient Protozoan, *Giardia lamblia*

**DOI:** 10.3389/fcimb.2019.00245

**Published:** 2019-07-24

**Authors:** Trevor T. Duarte, Cameron C. Ellis, Brian I. Grajeda, Atasi De Chatterjee, Igor C. Almeida, Siddhartha Das

**Affiliations:** ^1^Department of Biological Sciences, Border Biomedical Research Center, University of Texas at El Paso, El Paso, TX, United States; ^2^Infectious Disease and Immunology Cluster, Border Biomedical Research Center, University of Texas at El Paso, El Paso, TX, United States

**Keywords:** ceramide, cyst, encystation, *Giardia*, glycosphingolipids, sphingolipids, sphingomyelin, trophozoites

## Abstract

*Giardia lamblia*, a single-celled eukaryote, colonizes and thrives in the small intestine of humans. Because of its compact and reduced genome, *Giardia* has adapted a “minimalistic” life style, as it becomes dependent on available resources of the small intestine. Because *Giardia* expresses fewer sphingolipid (SL) genes—and glycosphingolipids are critical for encystation—we investigated the SL metabolic cycle in this parasite. A tandem mass spectrometry (MS/MS) analysis reveals that major SLs in *Giardia* include sphingomyelins, sphingoid bases, ceramides, and glycosylceramides. Many of these lipids are obtained by *Giardia* from the growth medium, remodeled at their fatty acyl chains and end up in the spent medium. For instance, ceramide-1-phosphate, a proinflammatory molecule that is not present in the culture medium, is generated from sphingosine (abundant in the culture medium) possibly by remodeling reactions. It is then subsequently released into the spent medium. Thus, the secretion of ceramide-1-phospate and other SL derivatives by *Giardia* could be associated with inflammatory bowel disease observed in acute giardiasis. Additionally, we found that the levels of SLs increase in encysting *Giardia* and are differentially regulated throughout the encystation cycle. We propose that SL metabolism is important for this parasite and, could serve as potential targets for developing novel anti-giardial agents.

## Introduction

Giardiasis is caused by an intestinal protist, *Giardia lamblia*, a leading cause of non-bacteria-associated diarrheal disease, resulting in an estimated 280 million annual cases worldwide (Ankarklev et al., 2010). Giardiasis is endemic in developing nations and mainly infects children. The symptoms of girdiasis are diarrhea, malabsorption, and malnutrition. Even after the parasite has been cleared, *Giardia* infection leads to a number of long-term disorders including failure to thrive, stunted growth, chronic fatigue, cognitive disorders, and irritable bowel syndrome (Halliez and Buret, 2013). The pathogen has a simple biphasic life cycle—i.e., replicative trophozoites and relatively dormant cysts (Adam, 2001). *Giardia* colonizes the upper intestinal lumen just below the bile duct where it multiplies through binary fission. While in the intestine, *Giardia* lives in an environment composed of mucus, bile salts, lipids, and fatty acids, and these components influence the growth, encystation, and excystation of the parasite (Gillin, 1987; Gillin et al., 1989).

Sphingolipids (SLs) are both important structural components of membranes and active signaling molecules that are implicated in various cellular processes such as growth, differentiation, apoptosis, senescence, and autophagy (Venable et al., 1995; Scarlatti et al., 2004). For example, while SLs were found to interact with growth-dependent signaling molecules in budding yeast (Clarke et al., 2017), their cellular accumulation in monogenic yeast caused mitochondrial dysfunction and cell death (Knupp et al., 2017). While early studies focused upon the role of entire SLs classes in cellular processes and disease states, current evidences suggest that each molecule, i.e., ceramide (Cer) and sphingomyelin (SM), is functionally distinct and regulate wide varieties of biological functions (Hannun and Obeid, 2008; Ben-David and Futerman, 2010; Grosch et al., 2012). Various classes of SLs accumulate in the midbodies and cleavage furrows of dividing mammalian cells (Atilla-Gokcumen et al., 2011). A recent report indicates that SLs are involved in maintaining a homeostatic balance of phosphoinositides (PIs) between trans-Golgi network and post-Golgi compartments, which is critical for Golgi-mediated cellular trafficking (Capasso et al., 2017). A previous study also suggests that SLs regulate the release of exosomes that promote the clearance of Aβ fibril associated with Alzheimer's disease (Yuyama et al., 2012).

*Giardia* is unique in the sense that only five SL metabolic genes have been annotated in the genome database (Morrison et al., 2007; Yichoy et al., 2011) and their functional identities are similar to eukaryotic SL enzymes (with low gene sequence identity). These five genes include two copies of giardial serine-palmitoyltransferases (i.e., *gspt-1* and *gspt-2*), one copy of giardial glucosylceramide transferase gene (*gglct1*), and two giardial acid sphingomyelinase-like phosphodiesterase genes (*gasmasepd B* and *gsmasepd 3b*). A transcriptomic analysis revealed that all five genes are transcribed and expressed differentially during the process of encystation and cyst production (Hernandez et al., 2008). Studies have also indicated that while serine-palmitoyltransferase (gSPT) enzymes regulate ceramide (Cer) endocytosis in *Giardia*, glucosylceramide transferase (gGlcT1) synthesizes glucosylceramide (GlcCer), which regulates giardial cell cycle, membrane trafficking, and encystation (Stefanic et al., 2010). Mendez et al. (2013) demonstrated that the overexpression and knockdown of the gGlcT1 enzyme in *Giardia* interferes with the biogenesis of encystation-specific vesicles (ESVs) and reduces the viability of cysts in culture. Interestingly, the normalization of gGlcT1 expression restores ESV biogenesis and cyst viability, further establishing the regulatory role of gGlcT1 in encystation and cyst production. More recently, it was demonstrated that gGlcT1 is a dual-substrate enzyme and can catalyze the synthesis of both GlcCer and galactosylceramide (GalCer) (Robles-Martinez et al., 2017). These results prompted us to analyze SLs in *Giardia* and the culture medium to elucidate the influence of environmental SLs in maintaining overall SL homeostasis in this parasite.

In the current study, we have carried out a targeted sphingolipidomic analysis by tandem mass spectrometry (MS/MS) of non-encysting, encysting, and water-resistant cysts of *Giardia* and compared it with the sphingolipidome of the culture medium. We found that: (i) sphingoid bases (sphingosine and sphinganine), ceramides (16 species), GSLs (40 different types), and sphingomyelin SM (18 species) are the predominant SLs in *Giardia* that mostly come from the culture medium; (ii) the levels of SLs are differentially regulated during encystation; (iii) ceramide-1-phosphate (Cer-1-P), is a newly generated lipid in this parasite; and (iv) *Giardia* has some ability to remodel exogenous SLs and release them back to the medium.

## Methods

### Chemicals and Antibodies

Unless otherwise specified, all chemicals were the highest purity available and purchased from Sigma-Aldrich (St. Louis, MO). All solvents used for mass spectrometry were of LC/MS grade. N-palmitoyl-d31-D-*erythro*-sphingosine (deuterated ceramide) and N-palmitoyl-d31-D-*erythro*-sphingosylphosphorylcholine (deuterated sphingomyelin), as well as all other SL standards, were obtained from Avanti Polar Lipids (Alabaster, AL). Fluorescein isothiocyanate (FITC)-conjugated trophozoite antibody (anti-rat polyclonal antibody; catalog no. A900; Troph-O-Glo) was purchased from Waterborne Inc. (New Orleans, LA). Mouse monoclonal cyst antibody and Alexa Fluor 568-conjugated donkey anti-mouse antibody was obtained from Santa Cruz (Santa Cruz, CA) and Invitrogen (Carlsbad, CA), respectively.

### Parasite and Encystation

*Giardia lamblia* trophozoites (assemblage A, strain WB C6; ATCC No. 30957) were cultivated following the method of Diamond et al. (1978) using modified TYI-S-33 medium supplemented with 10% adult bovine serum and 0.5 mg/ml bovine bile (Keister, 1983; Boucher and Gillin, 1990). The antibiotic piperacillin (100 μg/ml) was added during routine culturing of the parasite (Gillin et al., 1989). The growth was initiated by adding ~10^5^ trophozoites/ml in the culture medium and continued to grow until the cells became 80–90% confluent (~48 h). Parasites (~1 × 10^6^/ml) were harvested by detaching attached cells from the culture flask by ice chilling followed by centrifugation at 1,500 × g for 10 min at 4°C. Cell pellets were subjected to three washings in sterile phosphate-buffered saline (PBS) and microscopic determination of cell numbers (viability) using a hemocytometer (Mendez et al., 2013). *In vitro* encystation was performed by culturing the trophozoites in TYI-S-33 medium, pH 7.8, supplemented with adult bovine serum (10%, v/v), lactic acid (5 mM), and porcine bile (250 mg/ml) for various time points, as described previously (Gillin et al., 1989). Cells were allowed to encyst for 72 h, and cysts were isolated by centrifugation (2,500 × g for 10 min at 4°C), washed three times in cold, distilled water, and kept in water for 1-h at 4°C. Cells were immunostained with antibodies against trophozoite and cyst proteins as previously described (De Chatterjee et al., 2015).

### Lipid Extraction

*Giardia* trophozoites (2.5 × 10^6^ cells) were harvested and transferred to 13 x 150 mm-borosilicate glass tubes with polytetrafluoroethylene (PTFE) caps. Cells were washed twice with PBS and pelleted by centrifugation (1,500 × g, 5 min, 4°C). Parasites were resuspended in 1 ml PBS and, following removal of 100-μl aliquot for protein quantitation by Bradford assay, the remaining cells were lyophilized, flushed with N_2_, and stored at −80°C until extraction. This same procedure was followed for encysting cells and cysts. Lipids were also extracted from 1 ml adult bovine serum, adult bovine bile (52 mg), porcine bile (50 mg) and 1 ml of complete culture medium (fresh), as well as spent culture (SC) medium, following the same extraction procedure. Each liquid sample was lyophilized prior to extraction as with the cell pellets. Freeze-dried samples were resuspended in CHCl_3_: MeOH (2:1, v/v) and incubated at 48°C overnight. To remove possible interfering glycerophospholipids (GSLs), samples were treated with 100 mM (final concentration) KOH for 2-h at 37°C, then allowed to cool to room temperature and the pH was adjusted to 4–5 with glacial acetic acid (Sullards et al., 2007). Samples were centrifuged (900 × g, 5 min) and the supernatant was transferred to a clean tube. The remaining pellets were then extracted twice with isopropanol: hexane: H_2_O (55:25:20, v/v/v) and finally with CHCl_3_: MeOH (1:1, v/v) (Li et al., 2008). All four extracts were pooled and dried either under N_2_ stream or in a CentriVap Vacuum Centrifuge.

### Shotgun Sphingolipid Analysis by ESI-MS/MS

Due to the broad differences between classes of SLs, multiple separation techniques were followed as detailed below.

*Separation of neutral and acidic lipids by DEAE Chromatography*: DEAE-Cl resin (Sigma-Aldrich) was swelled overnight in 10 ml 0.5M Na-acetate in CHCl_3_: MeOH: H_2_O 30:60:8 (lower phase). Processed resin (1 ml) was then packed onto a Pasteur pipette over a small amount of glass wool. The resin was washed with 10 bed volumes (10 ml) CHCl_3_: MeOH: H_2_O (lower phase) and samples were loaded in the same solvent. Neutral lipids were eluted with 5 ml of the same solvent and acidic lipids were eluted with 5 ml of 0.8 M Na-acetate in MeOH. Eluates were dried as before, re-suspended in H_2_O and desalted using a 3 ml DSC-18 SPE cartridge (Supelco), dried and stored at −20°C under N_2_ until further processing. Neutral lipids were then per-N-acetylated with 4 ml acetic anhydride and 2 ml pyridine in the dark overnight then dried in centrivap.
*Enrichment of GSLS by Florisil Fractionation:* Florisil Chromatography was carried out to remove non-GSL impurities. Briefly, 300 mg of Florisil resin (60 mesh, Sigma-Aldrich) was packed into long-necked Pasteur Pipettes. The resin was washed with 10 ml dichloroethane (DCE): acetone 1:1 (v, v), then 10 ml DCE, and finally equilibrated with 10 ml hexane: DCE 1:4 (v, v). Samples were loaded in this solvent. Fraction F1 was eluted with 10 ml hexane: DCE 1:4 v, v; Fraction F2 was eluted with 10 ml DCE and Fraction F3, which is rich in GSLs, was eluted with DCE:acetone 1:1 v, v. Extracts were dried in centrivap and acetylated with 3 ml 0.5 N NaOMe in 6 ml MeOH for 2-h at room temperature. The reaction was stopped with 2 ml 10% acetic acid in MeOH (pH 4–5) desalted as mentioned above, dried and stored under N_2_ at −20°C.
*Per-N, O-Methylation of GSLS to increase ionization for MS:* Neutral GSL's from Florisil fraction F3 and acidic GSL's from DEAE eluate were methylated as described by Ciucanu (1984). Briefly GSLs were re-suspended in 150 μl DMSO, 40–50 mg of NaOH was added and stirred until dissolved. The reaction was started by adding 80 μl iodomethane and vortexed for 1- h at room temperature. The reaction was quenched with 2 ml H_2_O and GSLs were then extracted with dichloromethane (DCM). Extracts were washed with H_2_O, dried under N_2_, re-suspended to 1 mg/ml protein in MeOH and analyzed by nano-ESI mass spectrometry. Alkaline methanolysis to enrich for sphingoid bases (SBs) and sphingomyelins (SMs) as previously described by Jiang et al. (2007).

Analyses were carried out in positive ion mode on a linear triple quadrupole mass spectrometer (LTQ XL, Thermo Fisher Scientific) as described previously (Mendez et al., 2013). Total-ion mapping (TIM) from each fraction was used to determine the presence of SL species by analyzing the characteristic neutral loss or product-ion masses (Figures S1, S2). For example, methylated HexCer species produce a characteristic product ion at *m/z* 258.6 corresponding to [permethylated hexose + Na]^+^ (Ciucanu, 1984). Hex_2_Cer produces a peak at 463.4 [(permethylated hexose)_2_ + Na]^+^, and SM with a lithium adduct can be found with neutral loss of 59 [N(CH_3_)_3_]^+^ (Jiang et al., 2007) (For a complete list of TIM fragments used, see Table S1). For structural validation, putative SL species identified from ion mapping were fragmented further (MS3 to MS5 depending on analyte abundance) to confirm long-chain base (LCB) and fatty acyl (FA) chain length (Pernet et al., 2006). All files were acquired using Xcalibur software (Thermo Scientific).

### Quantitative Sphingolipid Analysis by TSQ Endura™ Triple Quadrupole MS

Samples from cell pellets (2.5 × 10^6^ cells) or culture medium were spiked with 500 pmol of N-palmitoyl-d31-D-*erythro*-sphingosine (deuterated ceramide) and N-palmitoyl-d31-D-*erythro*-sphingosylphosphorylcholine (deuterated sphingomyelin) as internal standards (expected response ratio 50 fmol/μl), extracted with CHCl_3_:MeOH (2:1, v/v), followed by isopropanol:hexane:H_2_O (55:25:20, v/v/v) and CHCl_3_:MeOH (1:1 v/v) (Li et al., 2008). Extracts were pooled, dried under the N_2_ stream, resuspended in MeOH (heated overnight at 48°C) and subjected to KOH hydrolysis. *HPLC:* Reverse-phase liquid chromatography was performed using a C18 column (Hypersil Gold 2.1 × 50 mm) in an UltiMate 3000 System (Dionex, Thermo Fisher Scientific). Solvent A: acetonitrile: water (30:70, v/v) mixed with 10 mM ammonium formate and 0.2% formic acid. Solvent B: isopropanol: acetonitrile (90:10) mixed with 10 mM ammonium formate and 0.2% formic acid. Samples were diluted 10–100 fold so that the response ratio of the highest peak fell within the linear range of a standard curve (Figure S3), and loaded into a column in 70/30 A/B. Gradient began at 30% B for 2 min followed by a ramp from 30% B to 70% B over 2 min, then ramped from 70% B to 99% B over 12 min, and plateaued at 99% B for 2 min with a return to starting conditions over 1 min. This was followed by equilibration for 10 min. *MRM:* SL quantitation was performed as previously described by Sullards et al. (2007). Samples were analyzed with a triple quadrupole mass spectrometer (TSQ Endura, Thermo Fisher Scientific) operating in the multiple-reaction monitoring (MRM) mode using mass lists identified above. MRM chromatograms were filtered for the exact mass of species of interest; peak detection and integration were performed using Xcalibur v2.1 processing software (ISCIS detection, smoothing 7, baseline window 40, area noise factor 10, peak noise factor 20, minimum peak height S/N 3). Integrated peak areas were divided by an integrated area of internal standard peak (d31-Cer for sphingosine (Sph), sphinganine (Spn), Cers, Cer-1-P, HexCers, Hex_2_Cers, and Hex_3_Cers; d31-SM for SMs) and the response ratio was plotted on linear calibration curves to provide the absolute concentration of SLs (Figure S3). Curves were generated by HPLC-MS/MS using increasing concentrations of calibration standards while maintaining 50 fmol/μl each d31-Cer and d31-SM, followed by peak integration, calculation of response ratio, and linear regression. Samples were analyzed in triplicate, and the results were recorded as mean plus standard error. SL species were confirmed by MS/MS fragmentation as well as retention time.

### Confocal Microscopy

To observe the expression of trophozoites-specific proteins, parasites were fixed in 4% paraformaldehyde for 15 min and immunostained with FITC-conjugated trophozoite antibody (Troph-O-Glo, anti-rat polyclonal antibody, Waterborne Inc., New Orleans, LA) as described by us earlier (De Chatterjee et al., 2015). For cyst-wall proteins, we used cyst antibody (1:100, monoclonal: Santa Cruz Biotechnology, Inc., Santa Cruz, CA). Alexa Fluor 568 donkey anti-mouse IgG (Invitrogen; 1:500 dilution) was used as a secondary antibody. ProLong® Gold antifade reagent with DAPI (Invitrogen) was used to visualize the nuclei. Images were captured by Carl Zeiss Laser Scanning Systems LSM 700 confocal microscopy. For intensity measurements, cells were selected randomly from 10 fields of 3–5 separate experiments. Approximately 50 cells were considered for each condition and were analyzed by Zeiss 2009 ZEN confocal software (De Chatterjee et al., 2015).

### Data Analysis

One-way ANOVA was used to compare the four treatment groups in each of 96 SL species identified. To alleviate the multiplicity issue, the false-discovery rate (FDR) was applied to make adjustment (Benjamini and Hochberg, 1995). Those comparisons with FDR-adjusted *p-*values smaller than α = 0.05 were deemed significant. When any statistically significant difference was found, pairwise comparison that compare each treatment vs. the “TROPH MEAN” group was conducted to gain further insight into the differences (Table S4). Paired *t*-test was applied to detect the pre-post difference in each of the outcome variables, which are 50 in total categorized into five groups. FDR was again used for multiplicity adjustment. One limitation of our analysis is that the small sample size (*n* = 3). Principal component analysis (PCA), pairwise student *t*-tests were performed using R (R Core Team, 2018). The confocal microscopy results were analyzed using Microsoft Excel (2007) (De Chatterjee et al., 2015) and were considered significant when *p* < 0.05.^*^

## Results

### Identification of Sphingolipid Species in *Giardia* by Shotgun Analysis

To identify SLs in *Giardia*, we initially used nano-electrospray ionization (nano-ESI) on a linear triple-quadrupole, ion-trap mass spectrometer (LTQ XL). Samples were subjected to per-*N*-methylation to identify GSL and Cer species. For the identification of sphingomyelins (SMs), samples were treated with lithium hydroxide (LiOH) before applying to nano-electrospray ionization-tandem mass spectrometry (ESI-MS/MS), as described in the Methods section. GSLs were identified by glycan ion fragments and Cer could be distinguished by a neutral loss of 48 atomic mass units (a.m.u.), as well as a prominent peak at *m/z* 278.6 (for d18:1 sphingoid base-containing species) (Table S1). Lithiated SM ([M+Li] ^+^) was detected by a neutral loss of 59 a.m.u. (Table S1). MS^n^ fragmentation to characterize the GSL and SM species (Figures S1, S2) was carried out following the methodology described by Levery (2005) and Hsu and Turk (2000). Thus, Cer, HexCer, Hex_2_Cer, Hex_3_Cer, SM, HexNAc-, and HexNAc-tetrahexosylceramide were identified as predominant neutral SLs in *Giardia* (Table S1). Interestingly, the majority of these species consisted of d18:1-sphingosine backbone and acyl chains ranging from C_14_ to C_24_. Acidic species identified were GM3, GD3 (d18:1/C14-C24), and GM1 (d18:1/16:0 and 18:0) (Table S2; Figures S2a,b); however, this method did not determine the stereo-configuration of the hexose residues. The result from this initial shotgun analysis encouraged us to carry out a targeted and quantitative analysis of SLs by multiple-reaction monitoring (MRM) using a triple-quadrupole mass spectrometer (TSQ Endura, Thermo Fisher Scientific), as previously described by Shaner et al. (2009).

### Quantitative Sphingolipid Analysis of *Giardia* and Its Culture Medium

By TSQ analysis, we identified 127 individual SL species (30 acidic and 97 neutral) (Table 1). Abundance of some of these lipids, such as Hex_3_Cers, were very low (below the level of quantification). Altogether, we were able to quantitate 7 of the 11 classes of lipids from cells and medium by the multiple-reaction monitoring (MRM) procedure, following the method previously described by Merrill et al. (2005). We found that SM species were the most abundant type of SL in *Giardia*, and such species were present with high abundancy in the culture medium (Table S3). Eighteen unique SM species were detected and their fatty acyl chains varied from 14 to 26 carbons. Interestingly, the majority (~88%) of SMs contained saturated fatty acids. Some SM species containing unsaturated (C24:1 and C23:1) acyl chains were also identified. Among saturated fatty acids, C16:0 accounted for ~40% SMs present in the medium and ~30% in trophozoites. In addition to C16:0, other abundant fatty acids conjugated to SMs in the medium, and cells were C23:0 followed by C18:0, C24:0, and C26:0 (Table S3).

**Table 1:** Total sphingolipid species identified and quantitated from *Giardia* and the medium by TSQ.

**Sphingolipids in *Giardia***	**All Species identified**	**Species quantitated**	**Species identified quantitated in culture medium**	**Species identified quantitated in spent medium**
**Species**	**Neutral**	**Numbers**	**Numbers**	**Numbers**
Sphingoid base	2	2	2	2
Cer-1-P	1	1	0	1
HexNAc-Hex_3_Cer	16	0	0	0
Hex_3_Cer	14	2	1	1
Hex_2_Cer	14	14	6	5
HexCer	16	16	6	6
Cer	16	16	16	16
SM	18	18	17	17
Total	97	68	48	48
	**Acidic**		**Acidic**	
SA-Hex_2_Cer	16	0	0	0
SA_2_-Hex_2_Cer	12	0	0	0
HexNAc-Hex_3_Cer	2	0	0	0
Total	30	0	0	0

Our analysis also revealed that Cers were the second-most abundant SLs after SMs, followed by sphingoid bases (SBs). A single species of Cer-1-P (d18:1/16:0) was found to be present in *Giardia* and SC medium but not in the growth medium. In case of the GSLs, Hex_2_Cer was an abundant species, followed by HexCer and small quantities of Hex_3_Cer in both the cells and the medium. The majority of GSLs from the cell extracts contained 18-carbon SBs and fatty acids ranging from 14to 26 carbons. Unsaturated fatty acids included C24:1 and C16:1 for HexCer, C24:1 for Hex_2_Cer, and both C23:1 and C24:1 for Hex_3_Cer. Unlike SM and Cer, GSL species were more diverse in *Giardia* than in the culture medium. Among GSLs, 7 species of HexCer, 6 species of Hex_2_Cer, and 1 species of Hex_3_Cer were identified in the medium as opposed to 14 types of Hex_2_Cer and Hex_3_Cer and 16 different classes of HexCer in *Giardia* (Tables S3, S4). As far as gangliosides are concerned, we were able to identify GM3 (SA-Hex2Cer), GD3 (SA2-Hex2Cer) and GM1 (SA-Hex4Cer) by per*-N*-methylation, but failed to quantify them by TSQ analysis. This could be due to the extraction procedure as we used reverse phase method instead of the normal phase extraction procedure. Analysis also showed that there was no dramatic differences of SL species in cells and the medium except the total number of lipids quantified in the medium were little less than *Giardia* cells (48 vs. 68) and Cer-1-P (one species) was only present in cells and the SC medium (Table 1).

### The Presence of a Dynamic Sphingolipid Metabolic Pathway

Although the growth medium (serum and bile) supplies the majority of SLs to *Giardia* (as discussed above), questions arise as to whether this parasite has the ability to alter/modify SLs and whether the lipids are recycled back to the medium. Earlier reports have suggested that *Giardia* utilizes deacylation/reacylation reactions to alter existing glycerol-based phospholipids (also known as the Land's cycle) and bypasses the synthesis of entirely new molecules via the *de novo* pathway (Das et al., 2001, 2002). Here, we examined the ability of *Giardia* to replace and modify the acyl chains of SLs.

**Figure 1:**
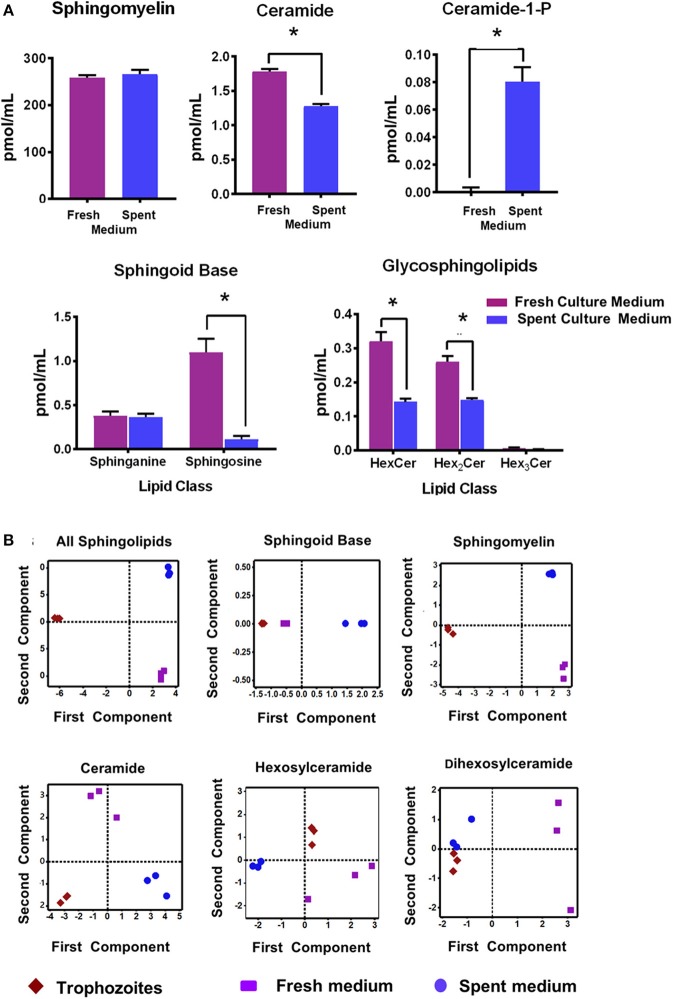
**(A)** Abundance of different classes of sphingolipids (SLs) in fresh and spent culture medium. Total abundances shown here are the sums of all long-chain base (LCB)/fatty acid (FA) species for (a) sphingomyelin (SM) (b) ceramide (Cer) (c) Cer-1-P (d) sphingosine bases (SBs), and (e) glycosphingolipids (GSLs). Concentrations of SLs are expressed in pmol/ml. *p < 0.05 by one-way ANOVA pairwise comparison after adjustment for false-discovery rate (FDR). **(B)** Principal component analysis (PCA) of SLs in *Giardia* trophozoites, fresh and spent culture medium. Analysis of the percent contribution of each long chain base/fatty acid (LCB/FA) composition to the total abundance of each class of lipid. In all cases. >98% differences were described by component 1. (a) All classes of SLs found in trophozoites, and fresh and spent culture medium. Each sample type maps to a unique quadrant indicating distinct sphingolipid profiles. This trend is visible in all lipid classes identified in the current study sphingomyelin (c), ceramide (d), hexosylceramide (e), and dihexosylceramide (f). The only exception appears to be the sphingoid bases sphinganine and sphingosine (b); the profile of which maps together for trophozoites and fresh culture medium and is distinct from the spent medium.

Figure 1A shows that the relative quantities of ceramides (Cer), sphingoild bases (SBs), and glycosphingolipids (GSLs) were lower in the spent culture (SC) medium when compared to the fresh medium (Figure 1A; Table S3). For example, sphinganine (Sph) was ~10 fold lower (1.1 vs. 0.11 pmol/ml) in the SC medium rather than the fresh (growth) medium, even though the level of sphingosine remained unchanged (Figure 1A “SBs”; 0.37 pmol/ml and 0.36 pmol/ml). Likewise, both Cers and GSLs were 30%−50% higher in the culture medium than in the SC medium, supporting the idea that majority of SLs were scavenged by *Giardia* from its medium. However, it appears that the amount of total sphingomyelin (SM) species in used and culture medium remain unaltered (Figure 1A). These results of SL profiles in fresh and SC medium encouraged us to examine their variability by a principal component analysis (PCA). We also included the SL results of trophozoites (Table S3) in our PCA analysis to examine the possible correlation of cellular lipids with fresh and SC medium.

Figure 1B demonstrates that the overall SL profiles (depicted as “all sphingolipids”) from trophozoites, culture, and SC medium were well-separated and partitioned in different quadrants, implying that their molecular composition could be different (Figure 1B; “all sphingolipids”). However, the exception to this finding was SBs. Sphingosine (Sph) was the major SB and the third-most abundant SL in both *Giardia* and medium, serving as a key backbone structure (83%−100%) of Cers, SMs, and GSLs (Tables S3, S4). Analysis of SBs (Figure 1B) reveals that while SBs from trophozoites and fresh culture medium occupied the same quadrant, they were quite different from SBs isolated from the SC medium. Analysis reveals that although SMs were highly abundant in both *Giardia* and the medium, they occupy separate quadrants, thus exhibiting their possible diversity in molecular compositions (Figure 1B). Cers from trophozoites and fresh and SC medium were also different from each other and positioned in separate quadrants as depicted in Figure 1B. Likewise, HexCers and dihexosylCers (Hex_2_Cer) from fresh and SC medium, as well as from trophozoites, were distinct from each other (Figure 1B). Hex_3_Cer was not included in this analysis, as its abundancy was beyond the detection level.

**Figure 2:**
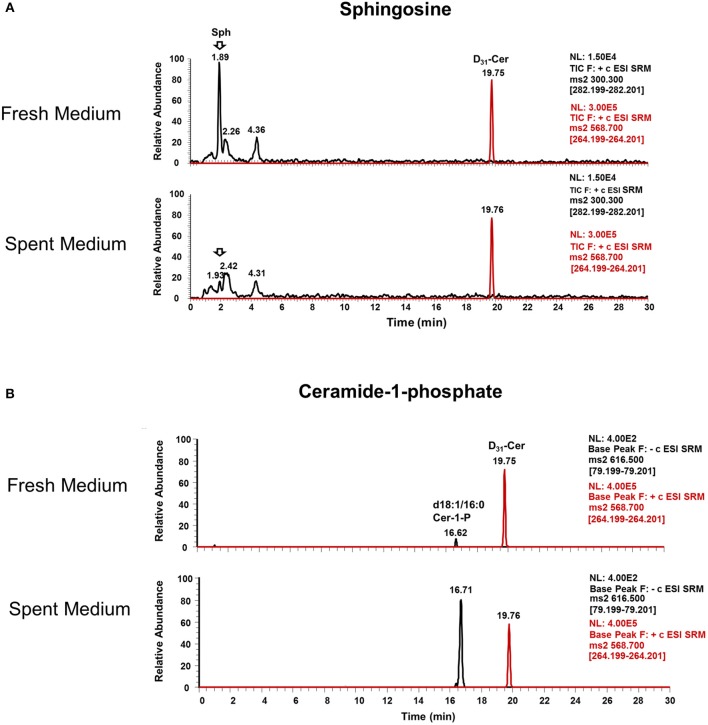
Ceramide-1-phosphate is a newly generated lipid in *Giardia*. **(A)** Representative MRM spectra used for relative quantitation of lipids in (a) fresh and (b) spent culture medium. The sphingosine (Sph) peak at 1.89 min is abundant in fresh medium (a) and nearly absent in spent medium (b), indicating the consumption of this lipid by trophozoites. **(B)** Representative MRM spectra used for relative quantitation of lipids in (c) fresh and (d) spent culture medium. Cer-1-P peak at 16.62 min in fresh medium (c) is very low compared to Cer-1-P at 16.71 min in (d), indicating the production of this class of lipid by *Giardia* trophozoites. The peak at 19.76 min is an internal standard (d_31_-Cer), added to each sample prior to lipid extraction.

**Figure 3:**
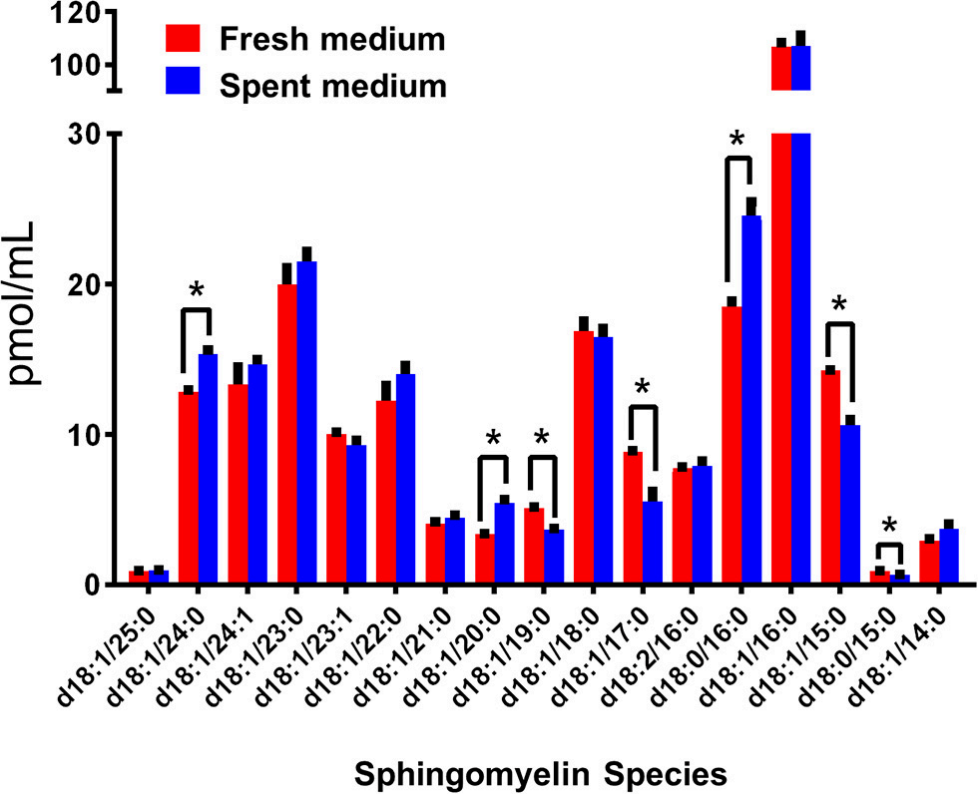
Sphingomyelin (SM) species in fresh (red) and spent (blue) culture medium. The species with significant changes in abundance indicate a trend in the production of even-numbered fatty acyl species and the consumption of odd chain fatty acyl species. The amount of individual SM is expressed in pmol/ml. *p < 0.05: Calculated by one-way ANOVA pairwise comparison after FDR adjustment.

Interestingly, Cer-1-P was not present in the fresh medium but detected in the SC medium (Figure 2A). We also analyzed the bovine serum and bile mixtures (both bovine and porcine) and failed to detect Cer-1-P (not shown), suggesting that this ceramide derivative is a newly generated lipid in this parasite and not comes from the medium. Further analysis indicated that Sph, which was abundant in fresh medium (Figure 2A), could serve as a precursor of Cer-1-P and released by a parasite into the medium (Figure 2B). Because the total amount of SMs in fresh and SC medium was similar (Figure 1A), we investigated the level of individual SMs and their distribution pattern. Out of all the SM species (Table S3), levels of three SMs containing odd-chain saturated fatty acids (d18:1/19:0, d18:1/17:0, and d18:1/15:0) were significantly higher in fresh medium. Conversely, three separate species containing even-chain saturated fatty acids (d18:1/24:0; d18:1/20:0; d18:0/16:0) were elevated in the SC medium. Three major SMs species (d18:1/18:0, d18:2/16:0, and d18:1/16:0) were equally distributed in fresh and SC medium. Although eight other minor SMs showed uneven distribution, the partitioning between the two different medium (fresh and SC) was not statistically significant (Figure 3).

### Changes of Sphingolipid Profile During Encystation

**Figure 4:**
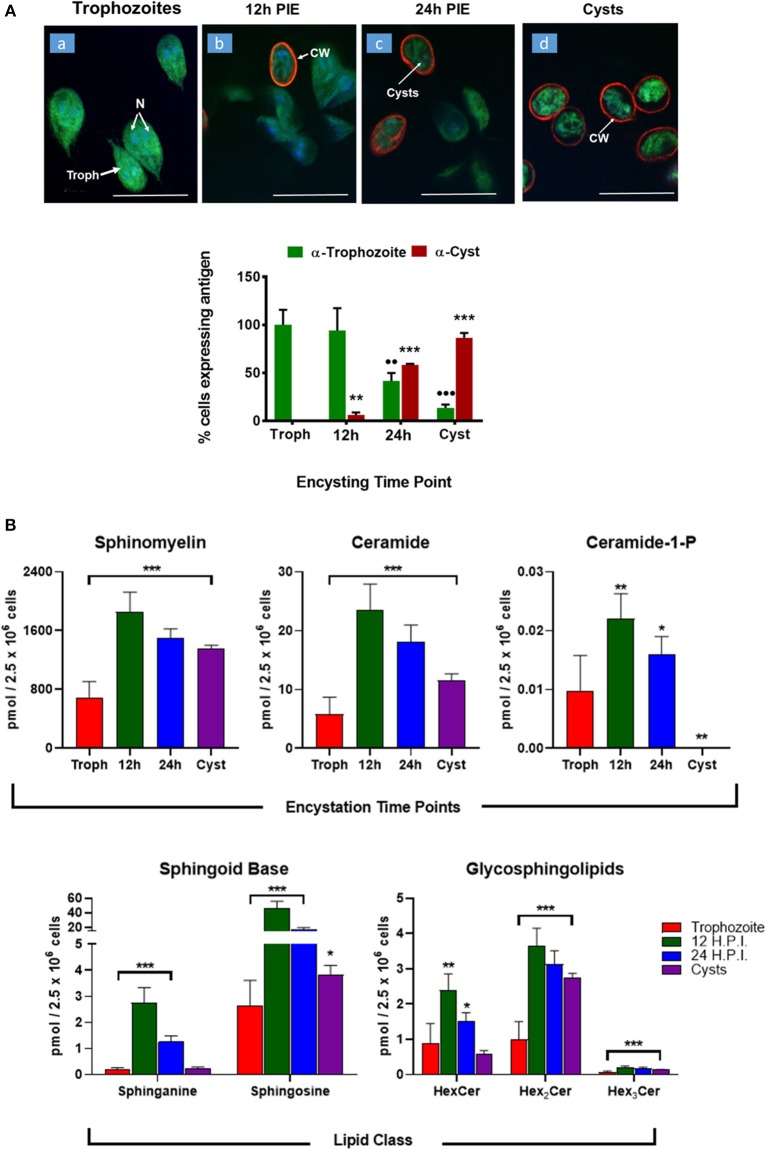
Expression of sphingolipids during encystation. **(A)** *In vitro* encystation and transformation of trophozoites to cysts was monitored by immunostaining with trophozoites and cyst antibodies. Images depict the percentage of cells expressing trophozoites (green) or cyst-wall (red) proteins at the various encysting time points used in this study (a, non-encysting trophozoites; b, 12-h PIE; c, 24-h PIE, and d, cysts). The expression of cyst proteins and cyst wall biogenesis were recorded as early as 12-h PIE (H.P.I) and continues to increase throughout encystation; *p < 0.05, **p < 0.01, ***p < 0.001, ^••^*p* < 0.01, ^•••^*p* < 0.001. **(B)** Abundance of SLs throughout giardial life cycle. Each graph represents the sum of all LCB/FA combinations for a given class during a specific time point of post-induction of encystation (PIE). Experiments were performed with equal number of cells (2.5 × 10^6^) and quantities of SLs are expressed in pmol. *p < 0.05, **p < 0.01, and ***p < 0.001: Calculated by one-way ANOVA followed by pairwise comparisons with trophozoites and FDR adjustment.

Encystation (or cyst formation) is an essential step of the *Giardia* life cycle. The induction of encystation triggers the biogenesis of cyst walls that covers the plasma membrane of the entire trophozoites (Lauwaet et al., 2007), as depicted in Figure 4A. Previously, it has been demonstrated that glucosylceramide transferase 1 (GlcT1)—an enzyme of the SL metabolic pathway—regulated the encystation and cyst production by *Giardia* (Mendez et al., 2013; Robles-Martinez et al., 2017), suggesting the importance of SLs in this process. Therefore, to gain an in-depth understanding regarding the abundance of SLs in encysting cells and cysts, we carried out *in vitro* encystation (Gillin et al., 1989) followed by extracting lipids from non-encysting trophozoites, encysting cells (12-h and 24-h post-induction of encystation or 12-h and 24-h PIE) and cysts and analyzed as described in the Methods section. It was noted that the level of SMs, GSLs (HexCer, Hex_2_Cer, and Hex_3_Cer), as well as Cer-1-P, increased ~2–3 fold at 12-h encysting cells (12-h PIE). Likewise, while the concentrations of Cer and Hex_2_Cer were elevated ~4 fold, the level of sphingosine increased ~16 fold compared to non-encysting trophozoites (Figure 4B, “sphingoid bases”). In each case, the level decreased in cysts but remained higher than non-encysting trophozoites, with the exception of Cer-1-P (Figure 4B, “Cer-1-P”), which was almost undetectable in cysts (Table S5). However, we must mention here that encysting cells (Figure 4A) used in this study were not synchronized and therefore not all cells were responsive to the encystation stimuli.

**Figure 5:**
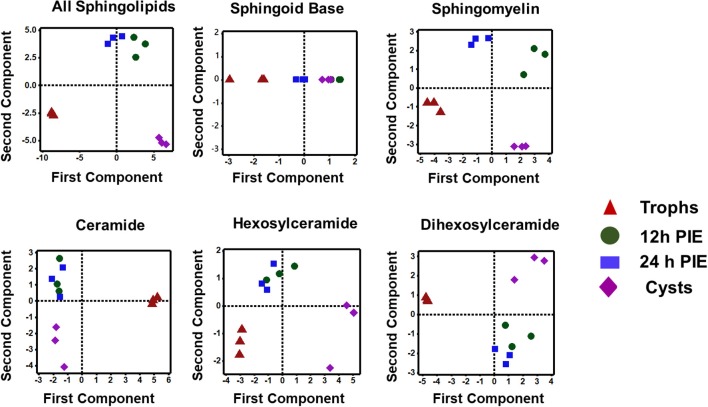
Principal component analysis of sphingolipids in *Giardia* during encystation. Analysis of the percent contribution of each long-chain base/fatty acid (LCB/FA) composition to the total abundance of each class of lipid. In all cases, >98% differences were described by component 1. (a) total sphingolipids extracted from non-encysting and encysting trophozoites (12-h PIE and 24-h PIE), as well as cysts. The samples map to three distinct quadrants, trophozoites, encysting cells, and cysts. This trend is visible in Cer, HexCer, and Hex_2_Cer (d-f), but not in SBs (b) and SM (c) when PCA is performed on each class of sphingolipid. In sphingomyelin (c), all four samples map to unique quadrants, whereas the SBs (b) are unresolved by encystation time-point.

The PCA analysis (Figure 5) revealed that SL species from trophozoites, encysting cells, and cysts formed a distinct pattern of distribution. However, it was observed that the encysting cells from two different time points (12- and 24-h PIE) clustered together, indicating that the molecular makeup of each SL in encysting cells could be different from trophozoites and cysts (Figure 5). These differences were clear in variations in the abundance of individual species and their contribution to the class as a whole. For GSLs, the differences could be attributed to the appearance of new GSLs during encystation (Mendez et al., 2013; Robles-Martinez et al., 2017) (Table S4; Figure 4B) and characteristic separation of encysting SLs from trophozoites and cysts (Figure 5). Specifically, as encystation progressed to form cysts, there was an increase in the species of Hex_2_Cer from ~1.0 pmol in trophozoites to ~3.0 pmol in cysts, while the total Hex_3_Cer abundance in cysts was only ~2 fold higher than trophozoites (Figure 4B, “glycosphingolipids”). Overall, the PCA identified a shift in the composition of SLs from trophozoites to encysting cells, as well as a change from encysting cells to cysts, indicating a unique distribution of SL species likely to be linked to the morphological changes that takes place at the time of stage differentiation from replicative trophozoites to relatively dormant cysts (Figure 5).

## Discussion

Parasitic cells are designed to scavenge lipids and other nutrients from their respective hosts and the surrounding environment. Gazos-Lopes et al. (2017) demonstrated that triacylglycerols (TGs) from host cells serve as major precursors of cellular lipids in *Trypanosoma cruzi* amastigotes and that blocking the supply of TGs interferes with their intracellular development. Lipidomic analysis reveals the presence of ether-type phospholipids in raft-domains of *Trypanosoma brucei* flagellar membranes, indicating their unique distributions and functions (Serricchio et al., 2015). Host-derived *lyso*-phosphatidylcholine (LPC) was shown to drive the biosynthesis of membrane lipids in the malaria parasite and also regulates its sexual differentiation (Brancucci et al., 2017). *Toxoplasma gondii*, which is capable of synthesizing the majority of its own SLs, recruits additional lipids from host cells by redirecting Golgi-derived Rab vesicles toward the parasitophorous vacuoles (Pratt et al., 2013; Romano et al., 2013). Likewise, the host-derived SM is critical for the maintenance of virulence of *Leishmania* within the host cell (Zhang et al., 2009). Sphingolipids and glucosylceramide also play critical role in fungal cell development (Del Poeta et al., 2014).

*Giardia* trophozoites colonize in the upper small intestine of humans and are thought to acquire the majority of their lipids from the dietary components of the intestinal milieu (Das et al., 1988; Stevens et al., 1997; Gibson et al., 1999). On the other hand, in culture medium, *Giardia* acquires it's lipids from bovine serum and bile (Yichoy et al., 2011). Phospholipids acquired by *Giardia* from the medium undergoes fatty acid and head group remodeling for the generation of new lipids by passing the synthesis via CDP-diacylglycerol (DAG) pathway (Subramanian et al., 2000; Das et al., 2001). Because SL genes are differentially expressed, and the activity of SL biosynthetic enzyme (gGlcT1/GalT) is upregulated during encystation (Hernandez et al., 2008; Mendez et al., 2013, 2015), our hypothesis is that SLs in *Giardia* are important for various biological functions and their cellular levels are well-coordinated with lipids present in the medium or small intestine.

Literature suggests that the analysis of cellular lipids by tandem MS is useful to examine the phenomenon of lipid homeostasis and changes of global lipid composition during the growth and differentiation of cells and organisms (Del Poeta et al., 2014; de Kroon, 2017). For example, ultra-high performance liquid chromatography-mass spectrometry (UHPLC-MS) has been useful to quantify more than 100 species of SLs in *Cordyceps* fungus and its related mycelia (Mi et al., 2016). In the current study, TSQ Endura Triple Quadrupole MS was employed to elucidate the metabolic profile of SLs in *Giardia* and demonstrate how that profile was influenced by the lipid pool in the medium.

We found that, although the overall SL profile in *Giardia* and the growth medium are similar (Tables S3, S4), the individual classes of lipids vary in fresh and SC medium (Figure 1A). The PCA analysis reveals that the molecular makeup of cellular SLs could be different compared to the medium, as they occupy different quadrants of the plot (Figure 1B), which could be due to the fact that length and saturation of acyl chains of SLs are diverse. Because the acyl chains of SLs in eukaryotic cells induce or repress autophagy and apopotosis (Clarke et al., 2017), and since the acyl chains of giardial SLs vary from C14 to C26, it is likely that both shorter and longer fatty acyl chains are critical in regulating various cellular functions, including signaling and differentiation. Furthermore, since the differences between SL bases and acyl chains are potentially important for inter-digitation and reduction in lateral diffusion (Niemela et al., 2006) the variations of acyl chain lengths and their respective distances from sphingoid bases could determine the flexibility of cyst membranes, which appears to be less dynamic than plasma membranes of trophozoites. Therefore, further investigation is essential to identify specifically favored combinations of fatty acyl chains of SLs and their possible effects on membrane biogenesis, encystation, and cyst production by *Giardia*.

Evidence shows that SLs and GSLs secreted by eukaryotic cells play an important role in maintaining cellular functions, cellular communications, and microbe-host cell interactions. In the case of sphingolipidosis (a pathogenic neuronal condition), brain cells release toxic SLs through exocytic vesicles to restore normal cellular functions (Scesa et al., 2016). Likewise, the white-rot fungus, *Ceriporiopsis subvermispora*, which degrades lignin, was shown to release cerebrosides to communicate with the surrounding microbial community (Nishimura et al., 2017). It is thus possible that *Giardia* uses the same strategy to communicate with microbial flora during colonization in the small intestine (Travers et al., 2016). Our results demonstrate that *Giardia* has the ability to generate new SLs by changing its fatty acyl moiety and subsequent release into the medium. For instance, Sph, an 18-carbon amino alcohol, is abundant in fresh medium, but it is not detected in SC medium (Figure 3). Conversely, Cer-1-P is present in spent, but not fresh medium (Figure 2). This suggests that Sph is scavenged by *Giardia* from the medium, converted first to Cer, then to Cer-1-P, and recycled back to the medium (Figures 2a,b). Likewise, the fatty acyl moieties of SMs also undergo remodeling reactions followed by secretion into the medium. For instance, SM species containing even-numbered fatty acids (i.e., d18:1/24:0, d18:1/20:0, and d18:0/16:0) are significantly higher in SC medium than fresh medium. In contrast, SMs containing long odd-chain, saturated fatty acids (d18:1/19:0, d18:1/17:0, and d18:1/15:0) are more abundant in fresh medium (Figure 3). These results imply that acyl chains could be a determinant factor for SM retention in cells. Retention or secretion or SMs could be important in regulating various cellular and metabolic functions both in parasite and host cells. Cer-1-P and other SLs are known to act as proinflammatory molecules that stimulate cytosolic phospholipase A_2_ activity with the subsequent release of arachidonic acid and eicosanoids (Presa et al., 2016). Reports suggest that *Giardia* secretes proteins during its interactions with colonic epithelial cells (Buret et al., 2015; Ma'ayeh et al., 2017; Coelho and Singer, 2018; Dubourg et al., 2018) and these proteins could considered as potential virulent factors. Our results suggest that *Giardia* also secretes lipids and may interfere with the host immune systems. Thus, it is possible that Cer-1-P and SMs secreted by *Giardia* trigger inflammatory bowel diseases that lead to a long-lasting pathophysiological condition in humans.

The process of cyst formation is critical for the survival of *Giardia* outside of the human body. Previously, it has been demonstrated that the inhibition of glucosylceramide (GlcCer) synthesis blocks endocytosis, cell cycle progression, biogenesis of encystation-specific vesicles (ESVs), and cyst viability (Stefanic et al., 2010; Mendez et al., 2013), which indicates that GlcCer and other SLs play an important role in cyst formation. Since the culture medium or the small intestinal milieu serve as a major source of SLs in *Giardia*, we examined how the parasite alters exogenous lipids during the process of stage-specific differentiation to cysts. Our results clearly demonstrate that majority of SL species tested in this investigation are higher in encysting cells and cysts than non-encysting trophozoites (Figure 4). Furthermore, these SL species appear to be different in trophozoites, encysting cells, and cysts (Figure 5). This is interesting because in a previous study (Duarte, 2014), we observed that the overall SL species in culture and encystation medium did not differ significantly. Therefore, it is likely that *Giardia* has some ability to alter/remodel lipid species in trophozoites and throughout the encystation process.

Stage-specific expression of SLs may have long-term biological implications in *Giardia*. Increased Cer (Figure 4B) is likely to be involved in synthesizing new GSLs facilitated by giardial GlcT1 (Mendez et al., 2013; Robles-Martinez et al., 2017). Although GSLs are acquired by *Giardia* from the medium, it remains unclear how this parasite selects and utilizes both endogenous and exogenous SLs for the purpose of cell signaling and encystation (Figure 4B). As is known, SLs are important components of *Giardia* membrane microdomains (De Chatterjee et al., 2015) and Cers and GlyCers are established signaling molecules involved in diverse cellular functions from differentiation to apoptosis (Ishibashi et al., 2013). Elevation of these lipids during encystation indicates a precise role of SL signaling during cyst formation or, more specifically, a possible structural alteration of the plasma membrane required for deposition of cyst wall material. Likewise, an increase in the SM concentration in encysting cells could also have the reverse effect on plasma membrane integrity/function. SM is an abundant lipid at the membrane; in combination with cholesterol and protein, it can form lipid rafts. An increase in SM (Figure 4B) could affect the membrane by altering the temperature at which it is liquid- or solid-ordered, as has been shown in model membrane systems with SM and cholesterol (Bartels et al., 2008). High SM concentrations could be required to stabilize the membrane for cysts in an outer environment in which temperatures are substantially lower than temperatures in the small intestine of humans. Sphingoid bases, which are known to act as signaling molecules (Cannavo et al., 2017) and membrane stabilizers (Carreira et al., 2017) are also upregulated in encysting cells and cysts, suggesting that they effectively participate in the encystation signaling process. Sphingosine also acts as a precursor of Cer-1-P in *Giardia*, which is secreted by this parasite (Figure 2B), and likely to be involved during the host-*Giardia* interactions. In case of GSLs, we observed that there are more GSL species (HexCer, Hex_2_Cer, and Hex_3_Cer) in trophozoites and encysting cells than in the medium (Tables S3, S4). The abundance of GSL species, especially mono-hexosyl and di-hexosylceramides are elevated during early encysting cells (12-h PIE) and declines during late encystation (24-h PIE) and in cysts (Figure 4). This can be attributed to the fact that gGlcT1 activity is upregulated in encysting cells and could therefore be linked to the increased synthesis of new GSLs (Mendez et al., 2013; Robles-Martinez et al., 2017). Interestingly, in an earlier report, Stefanic et al. (2010) demonstrated that Hex3Cer level in *Giardia* increases during encystation and its abundance in encysting cells is several fold higher than non-encysting trophozoites or other GSLs. At this point it is not clear why our results are different than that of Stefanic et al. (2010), however it is possible that we have used different instruments and/or separate extraction procedures. Furthermore, we have expressed our results relative to cell numbers (Figure 4) rather than the protein concentrations because we found that the amount of proteins are less in cysts compared trophozoites (not shown).

In summary, we have demonstrated that *Giardia* has evolved a unique strategy to import and utilize exogenous SLs for various cellular functions, including the assembly of membrane rafts and as mediators of the encystation induction pathway (De Chatterjee et al., 2015; Mendez et al., 2015). Our sphingolipidomic analysis support the notion that *Giardia* uses the acyl chain remodeling/alteration pathway to generate new SL species by passing the synthesis of entirely new lipid molecules. However, the genome search so far failed to identify ceramide kinase and other lipid remodeling genes, suggesting the presence of unusual lipid metabolic pathways in this ancient protist. Our future goal will be to continue our search using the combination of bioinformatics and molecular tools as previously reported (Robles-Martinez et al., 2017; Ye et al., 2017). It is likely that some of the SL genes that exist in *Giardia* are sufficiently diverged from their mammalian hosts, thus enabling researchers to target in designing future anti-giardial drugs.

## Author Contributions

TD, IA, and SD designed the research. TD performed the research. CE and BG were involved in repeating some of the experiments and collaborated with TD in manuscript data acquisition and statistical analysis. TD and SD analyzed the data and wrote the paper. IA examined and commented on the data analysis and helped to complete the manuscript. AD was involved in carrying out the microscopic experiments and analyzing results.

### Conflict of Interest Statement

The authors declare that the research was conducted in the absence of any commercial or financial relationships that could be construed as a potential conflict of interest.

## Supplementary Material

Figure S1 Representative MS^*n*^ fragmentation analysis with assignment of Cer and LCB. **(a)** *m/z* 806.5, Per-*N, O*-methylated d18:1/16:0-HexCer with Na^+^ adduct. **(b)** *m/z* 1010.5, Per-*N, O*-methylated d18:1/16:0-Hex_2_Cer with Na^+^ adduct. **(c)** *m/z* 1214.8, Per-*N, O*-methylated d18:1/16:0- Hex_2_Cer with Na^+^ adduct. **(d)** *m/z* 1459.9, Per-*N, O*-methylated d18:1/16:0-HexNAc-Hex_3_Cer with Na^+^ adduct.

Figure S2 Representative MS^*n*^ fragmentation analysis with assignment of Cer and LCB. **(a)** *m/z* 1372.7, Per-*N, O*-methylated d18:1/16:0-monosialo-Hex_2_Cer (GM_3_) with Na^+^ adduct. **(b)** *m/z* 1733.2, Per-N, O-methylated d18:1/16:0-disialo-Hex_2_Cer (GM3) with Na^+^ adduct. **(c)** *m/z* 709.6, d18:1/16:0-SM with Li^+^ adduct. **(d)** *m/z* 807.7, d18:1/23:0-SM with Li^+^ adduct.

Figure S3 Calibration curves used for quantitation of sphingolipids. For each class, 4–7 concentrations were used to construct the curve. The graph is the plot of the response ratio between varying concentrations of calibration standards and 50 fmol/μl internal standards. For internal standards, both deuterated and non-deuterated (d18:1/12.0 and d18:1/8.0) lipids were used. Equations and R^2^ values generated were used to transform response ratios to concentration values for each sample. **(A)** (a) d18:1/12:0-, d18:1/16:0-, and d18:1/24:1-SM; (b) d17:1/16:0- and d18:1/24:1-Cer; (c) d18:1/12:0-, d18:1/16:0-, and d18:1/24:1-glucosylceramide (GlcCer); (d) d18:1/8:0-, d18:1/16:0-, and d18:1/C24:1-lactosylceramide (LacCer); d18:1/16:0-Cer-1-P; and d18:1-Sph. **(B)** Standard ratio equations and *R*^2^ values.

Table S1 Neutral sphingolipid species identified by direct infusion nano-ESI MS/MS using TIM and MS^n^ fragmentation.

Table S2 Acidic sphingolipids identified by direct infusion nano-ESI MS/MS using TIM and MS^*n*^ fragmentation.

Table S3 Absolute concentration (pmol/ml) of sphingolipids in fresh and spent culture medium. Statistical analysis was carried out by one-way ANOVA pairwise comparison of media before (fresh) and after (spent) culture with giardial trophozoite cells.

Table S4 Absolute concentration (pmol/2.5 × 10^6^ cells) of individual species of sphingolipids throughout the encystation cycle. Statistical analysis was performed by one-way ANOVA between the four life cycle stages (trophozoite, 12-h PIE, 24-h PIE, and cysts) used in the current study. Statistical analysis was carried out by one-way ANOVA pairwise comparison and FDR adjustment. Percentage of each classes of sphingolipid and their respective standard deviations are also shown.

Table S5 Changes sphingolipid species in non-encysting and encysting trophozoites as well as in water-resistant cysts. Results are shown in percentages and errors (±SD) for each class of sphingolipids.

## Abbreviations

Cer: ceramide
Cer-1-P: ceramide-1-phosphate
GlyCer: glycosylceramide
GSL: glycosphingolipid
LCB/FA: long chain base/fatty acid
LTQXL: linear triple quadrupole ion-trap mass spectrometer
MRM: multiple reaction monitoring
PIE: post-induction of encystation
SA: sialic acid
SC medium: spent culture medium
SB: sphingoid bases
SL: sphingolipid
SM: sphingomyelin
Sph: sphingosine
Spn: sphinganine
TIM: total-ion mapping
TSQ: triple quadrupole mass spectrometer.

## References

[B1] Adam R. D. (2001). Biology of *Giardia lamblia*. Clin. Microbiol. Rev. 14, 447–475. 10.1128/CMR.14.3.447-475.2001 11432808 PMC88984

[B2] Ankarklev J. Jerlstrom-Hultqvist J. Ringqvist E. Troell K. Svard S. G. (2010). Behind the smile: cell biology and disease mechanisms of *Giardia* species. Nat. Rev. Microbiol. 8, 413–422. 10.1038/nrmicro2317 20400969

[B3] Atilla-Gokcumen G. E. Bedigian A. V. Sasse S. Eggert U. S. (2011). Inhibition of glycosphingolipid biosynthesis induces cytokinesis failure. J. Am. Chem. Soc. 133, 10010–10013. 10.1021/ja202804b 21668028 PMC3131740

[B4] Bartels T. Lankalapalli R. S. Bittman R. Beyer K. Brown M. F. (2008). Raftlike mixtures of sphingomyelin and cholesterol investigated by solid-state 2H NMR spectroscopy. J. Am. Chem. Soc. 130, 14521–14532. 10.1021/ja801789t 18839945 PMC2756786

[B5] Ben-David O. Futerman A. H. (2010). The role of the ceramide acyl chain length in neurodegeneration: involvement of ceramide synthases. Neuromol. Med. 12, 341–350. 10.1007/s12017-010-8114-x 20502986

[B6] Benjamini Y. Hochberg Y. (1995). Controlling the false discovery rate: a practical and powerful approach to multiple testing. J. R. Stat. Soc. B. 57, 289–300. 10.1111/j.2517-6161.1995.tb02031.x

[B7] Boucher S. E. Gillin F. D. (1990). Excystation of *in vitro*-derived *Giardia lamblia* cysts. Infect. Immun. 58, 3516–3522. 2228222 10.1128/iai.58.11.3516-3522.1990 PMC313691

[B8] Brancucci N. M. B. Gerdt J. P. Wang C. De Niz M. Philip N. Adapa S. R.. (2017). lysophosphatidylcholine regulates sexual stage differentiation in the human malaria parasite *Plasmodium falciparum*. Cell 171, 1532–1544. 10.1016/j.cell.2017.10.020 29129376 PMC5733390

[B9] Buret A. G. Amat C. B. Manko A. Beatty J. K. Halliez M. C. M. Bhargava A. (2015). *Giardia duodenalis*: new research developments in pathophysiology, pathogenesis, and virulence factors. Curr. Trop. Med. Rep. 2, 110–118. 10.1007/s40475-015-0049-8

[B10] Cannavo A. Liccardo D. Komici K. Corbi G. de Lucia C. Femminella G. D.. (2017). Sphingosine kinases and sphingosine 1-phosphate receptors: signaling and actions in the cardiovascular system. Front. Pharmacol. 8:556. 10.3389/fphar.2017.00556 28878674 PMC5572949

[B11] Capasso S. Sticco L. Rizzo R. Pirozzi M. Russo D. Dathan N. A.. (2017). Sphingolipid metabolic flow controls phosphoinositide turnover at the trans-Golgi network. EMBO J. 36, 1736–1754. 10.15252/embj.201696048 28495678 PMC5470045

[B12] Carreira A. C. de Almeida R. F. M. Silva L. C. (2017). Development of lysosome-mimicking vesicles to study the effect of abnormal accumulation of sphingosine on membrane properties. Sci. Rep. 7:3949. 10.1038/s,41598-017-04125-6 28638081 PMC5479847

[B13] Ciucanu F. K (1984). Simple and rapid method for the permethylation of carbohydrates. Carbohydr. Res. 131, 209–217. 10.1016/0008-6215(84)85242-8

[B14] Clarke J. Dephoure N. Horecka I. Gygi S. Kellogg D. (2017). A conserved signaling network monitors delivery of sphingolipids to the plasma membrane in budding yeast. Mol. Biol. Cell. 28, 2589–2599. 10.1091/mbc.e17-01-0081 28794263 PMC5620368

[B15] Coelho C. H. Singer S. M. (2018). Recent advances in the *Giardia*–host relationship reveal danger lurking behind the smile. PLoS Negl. Trop. Dis. 12:e0006625. 10.1371/journal.pntd.0006625 30188894 PMC6126833

[B16] Das S Stevens T. Castillo C. Villasenõr A. Arredondo H. Reddy K. (2002). Lipid metabolism in mucous-dwelling amitochondriate protozoa. Int. J. Parasitol. 32, 655–675. 10.1016/S.0020-7519(02)00006-1 12062485

[B17] Das S. Castillo C. Stevens T. (2001). Phospholipid remodeling/generation in *Giardia*: the role of the lands cycle. Trends Parasitol. 17, 316–319. 10.1016/S1471-4922(01)01901-8 11423372

[B18] Das S. Reiner D. S. Zenian J. Hogan D. L. Koss M. A. Wang C. S.. (1988). Killing of *Giardia lamblia* trophozoites by human intestinal fluid *in vitro*. J Infect Dis. 157, 1257–1260. 10.1093/infdis/157.6.1257 3373026

[B19] De Chatterjee A. Mendez T. L. Roychowdhury S. Das S. (2015). The assembly of GM1 glycolipid and cholesterol-enriched raft-like membrane microdomains is important for giardial encystation. Infect. Immun. 83, 2030–2042. 10.1128/IAI.03118-14 25733521 PMC4399036

[B20] de Kroon A. I. P. M. Lipidomics in research on yeast membrane lipid homeostasis. (2017). Biochim. Biophys. Acta 1862, 797–799. 10.1016/j.bbalip.2017.02.007 28219720

[B21] Del Poeta M. Nimrichter L. Rodrigues M. L. Luberto C. (2014). Synthesis and biological properties of fungal glucosylceramide. PLoS Pathog. 10:e1003832. 10.1371/journal.ppat.1003832 24415933 PMC3887071

[B22] Diamond L. S. Harlow D. R. Cunnick C. C. (1978). A new medium for the axenic cultivation of *Entamoeba histolytica* and other *Entamoeba*. Trans. R. Soc. Trop. Med. Hyg. 72, 431–432. 10.1016/0035-9203(78)90144-X 212851

[B23] Duarte T. (2014). Global Sphingolipid Profile of Giardia lamblia During Stage Differentiation: The Influence of Sphingomyelin Abundance on Cyst Viability. El Paso, TX: ETD Collection for University of Texas. Available online at: https://digitalcommons.utep.edu/dissertations/AAI3636253

[B24] Dubourg A. Xia D. Winpenny J. P. Al Naimi S. Bouzid M. Sexton D. W.. (2018). Giardia secretome highlights secreted tenascins as a key component of pathogenesis. Gigascience 7, 1–13. 10.1093/gigascience/giy,003 29385462 PMC5887430

[B25] Gazos-Lopes F. Martin J. L. Dumoulin P. C. Burleigh B. A. (2017). Host triacylglycerols shape the lipidome of intracellular trypanosomes and modulate their growth. PLoS Pathog. 13:e1006800. 10.1371/journal.ppat.1006800 29281741 PMC5760102

[B26] Gibson G. R. Ramirez D. Maier J. Castillo C. Das S. (1999). *Giardia lamblia*: incorporation of free and conjugated fatty acids into glycerol-based phospholipids. Exp. Parasitol. 92, 1–11. 10.1006/expr.1999.4389 10329359

[B27] Gillin F. D. (1987). *Giardia lamblia*: the role of conjugated and unconjugated bile salts in killing by human milk. Exp. Parasitol. 63, 74–83. 10.1016/0014-4894(87)90080-4 3803534

[B28] Gillin F. D. Boucher S. E. Rossi S. S. Reiner D. S. (1989). *Giardia lamblia*: the roles of bile, lactic acid, and pH in the completion of the life cycle *in vitro*. Exp. Parasitol. 69, 164–174. 10.1016/0014-4894(89)90185-9 2753120

[B29] Grosch S. Schiffmann S. Geisslinger G. (2012). Chain length-specific properties of ceramides. Prog. Lipid Res. 51, 50–62. 10.1016/j.plipres.2011.11.001 22133871

[B30] Halliez M. C. Buret A. G. (2013). Extra-intestinal and long term consequences of *Giardia* duodenalis infections. World J. Gastroenterol. 19, 8974–8985. 10.3748/wjg.v19.i47.8974 24379622 PMC3870550

[B31] Hannun Y. A. Obeid L. M. (2008). Principles of bioactive lipid signalling: lessons from sphingolipids. Nat. Rev. Mol. Cell. Biol. 9, 139–150. 10.1038/nrm2329 18216770

[B32] Hernandez Y. Shpak M. Duarte T. T. Mendez T. L. Maldonado R. A. Roychowdhury S.. (2008). Novel role of sphingolipid synthesis genes in regulating giardial encystation. Infect. Immun. 76, 2939–2949. 10.1128/IAI.00116-08 18426892 PMC2446683

[B33] Hsu F. F. Turk J. (2000). Structural determination of sphingomyelin by tandem mass spectrometry with electrospray ionization. J. Am. Soc. Mass Spectrom. 11, 437–449. 10.1016/S1044-0305(99)00150-6 10790848

[B34] Ishibashi Y. Kohyama-Koganeya A. Hirabayashi Y. (2013). New insights on glucosylated lipids: metabolism and functions. Biochim. Biophys. Acta 1831, 1475–1485. 10.1016/j.bbalip.2013.06.001 23770033

[B35] Jiang X. Cheng H. Yang K. Gross R. W. Han X. (2007). Alkaline methanolysis of lipid extracts extends shotgun lipidomics analyses to the low-abundance regime of cellular sphingolipids. Anal. Biochem. 371, 135–145. 10.1016/j.ab.2007.08.019 17920553 PMC2131739

[B36] Keister D. B. (1983). Axenic culture of *Giardia lamblia* in TYI-S-33 medium supplemented with bile. Trans. R Soc. Trop. Med. Hyg. 77, 487–488. 10.1016/0035-9203(83)90120-7 6636276

[B37] Knupp J. Martinez-Montañés F. Van Den Bergh F. Cottier S. Schneiter R. Beard D.. (2017). Sphingolipid accumulation causes mitochondrial dysregulation and cell death. Cell Death Differ. 24, 2044–2053. 10.1038/cdd.2017.128 28800132 PMC5686345

[B38] Lauwaet T Davids B. J. Reiner D. S. Gillin F. D. (2007). Encystation of *Giardia lamblia*: a model for other parasites. Curr. Opin. Microbiol. 10, 554–559. 10.1016/j.mib.2007.09.011 17981075 PMC2709507

[B39] Levery S. B. (2005). Glycosphingolipid structural analysis and glycosphingolipidomics. Methods Enzymol. 405, 300–369. 10.1016/S0076-6879(05)05012-3 16413319

[B40] Li Y. Teneberg S. Thapa P. Bendelac A. Levery S. B. Zhou D. (2008). Sensitive detection of isoglobo and globo series tetraglycosylceramides in human thymus by ion trap mass spectrometry. Glycobiology 18, 158–165. 10.1093/glycob/cwm129 18056651

[B41] Ma'ayeh S. Y. Liu J. Peirasmaki D. Hörnaeus K. Bergström Lind S. Grabherr M.. (2017). Characterization of the Giardia intestinalis secretome during interaction with human intestinal epithelial cells: the impact on host cells. PLoS Negl. Trop. Dis. 11:e0006120. 10.1371/journal.pntd.0006120 29228011 PMC5739509

[B42] Mendez T. L. De Chatterjee A. Duarte T. De Leon J. Robles-Martinez L. Das S. (2015). Sphingolipids, lipid rafts, and giardial encystation: the show must go on. Curr. Trop. Med. Rep. 2, 136–143. 10.1007/s40475-015-0052-0 26587369 PMC4646163

[B43] Mendez T. L. De Chatterjee A. Duarte T. T. Gazos-Lopes F. Robles-Martinez L. Roy D.. (2013). Glucosylceramide transferase activity is critical for encystation and viable cyst production by an intestinal protozoan, *Giardia lamblia*. J. Biol. Chem. 288, 16747–16760. 10.1074/jbc.M112.438416 23589290 PMC3675608

[B44] Merrill A. H. Jr. Sullards M. C. Allegood J. C. Kelly S. Wang E. (2005). Sphingolipidomics: high-throughput, structure-specific, and quantitative analysis of sphingolipids by liquid chromatography tandem mass spectrometry. Methods 36, 207–224. 10.1016/j.ymeth.2005.01.009 15894491

[B45] Mi J. N. Wang J. R. Jiang Z. H. (2016). Quantitative profiling of sphingolipids in wild Cordyceps and its mycella by using UHPLC-MS. Sci. Rep. 6:20870. 10.1038/srep20870 26868933 PMC4751452

[B46] Morrison H. G. McArthur A. G. Gillin F. D. Aley S. B. Adam R. D. Olsen G. J.. (2007). Genomic minimalism in the early diverging intestinal parasite Giardia lamblia. Science 317, 1921–1926. 10.1126/science.1143837 17901334

[B47] Niemela P. S. Hyvonen M. T. Vattulainen I. (2006). Influence of chain length and unsaturation on sphingomyelin bilayers. Biophys J. 90, 851–863. 10.1529/biophysj.105.067371 16284257 PMC1367110

[B48] Nishimura H. Yamaguchi D. Watanabe T. (2017). Cerebrosides, extracellular glycolipids secreted by the selective lignin-degrading fungus *Ceriporiopsis subvermispora*. Chem. Phys. Lipids 203, 1–11. 10.1016/j.chemphyslip.2016.12.006 28062355

[B49] Pernet F. Pelletier C. J. Milley J. (2006). Comparison of three solid-phase extraction methods for fatty acid analysis of lipid fractions in tissues of marine bivalves. J. Chromatogr. A 1137, 127–137. 10.1016/j.chroma.2006.10.059 17097094

[B50] Pratt S. Wansadhipathi-Kannangara N. K. Bruce C. R. Mina J. G. Shams-Eldin H. Casas J.. (2013). Sphingolipid synthesis and scavenging in the intracellular apicomplexan parasite, *Toxoplasma gondii*. Mol. Biochem. Parasitol. 187, 43–51. 10.1016/j.molbiopara.2012.11.007 23246819 PMC3629565

[B51] Presa N. Gomez-Larrauri A. Rivera I. G. Ordoñez M. Trueba M. Gomez-Muñoz A. (2016). Regulation of cell migration and inflammation by ceramide 1-phosphate. Biochim. Biophys. Acta 186, 402–409. 10.1016/j.bbalip 26875839

[B52] R Core Team (2018). R: A Language and Environment for Statistical Computing. Vienna: R Foundation for Statistical Computing. Available online at: https://www.R-project.org (accessed April 29, 2019).

[B53] Robles-Martinez L. Mendez T. L. Apodaca J. Das S. (2017). Glucosylceramide transferase in Giardia preferentially catalyzes the synthesis of galactosylceramide during encystation. Mol. Biochem. Parasitol. 211, 75–83. 10.1016/j.molbiopara.2016.11.001 27840079 PMC5222682

[B54] Romano J. D. Sonda S. Bergbower E. Smith M. E. Coppens I. (2013). Toxoplasma gondii salvages sphingolipids from the host Golgi through the rerouting of selected Rab vesicles to the parasitophorous vacuole. Mol. Biol. Cell. 24, 1974–1995. 10.1091/mbc.e12-11-0827 23615442 PMC3681701

[B55] Scarlatti F. Bauvy C. Ventruti A. Sala G. Cluzeaud F.. (2004). Ceramide-mediated macroautophagy involves inhibition of protein kinase B and up-regulation of beclin 1. J. Biol. Chem. 279, 18384–18391. 10.1074/jbc,.M.313561200 14970205

[B56] Scesa G. Moyano A. L. Bongarzone E. R. Givogri M. I. (2016). Port-to-port delivery: mobilization of toxic sphingolipids via extracellular vesicles. J. Neurosci. Res. 94, 1333–1340. 10.1002/jnr.23798 27638615 PMC5027965

[B57] Serricchio M. Schmid A. W. Steinmann M. E. Sigel E. Rauch M. Julkowska D.. (2015). Flagellar membranes are rich in raft-forming phospholipids. Biol. Open 4, 1143–1153. 10.1242/bio.011957 26276100 PMC4582118

[B58] Shaner R. L. Allegood J. C. Park H. Wang E. Kelly S. Haynes C. A.. (2009). Quantitative analysis of sphingolipids for lipidomics using triple quadrupole and quadrupole linear ion trap mass spectrometers. J. Lipid Res. 50, 1692–1707. 10.1194/jlr.D800051-JLR200 19036716 PMC2724058

[B59] Stefanic S. Spycher C. Morf L. Fabrias G. Casas J.. (2010). Glucosylceramide synthesis inhibition affects cell cycle progression, membrane trafficking, and stage differentiation in *Giardia lamblia*. J. Lipid Res. 51, 2527–2545. 10.1194/jlr.M003392 20335568 PMC2918437

[B60] Stevens T. L. Gibson G. R. Adam R. Maier J. Allison-Ennis M. Das S. (1997). Uptake and cellular localization of exogenous lipids by *Giardia lamblia*, a primitive eukaryote. Exp. Parasitol. 86, 133–143. 10.1006/expr.1997.4162 9207743

[B61] Subramanian A. B. Navarro S. Carrasco R. A. Marti M. Das S. (2000). Role of exogenous inositol and phosphatidylinositol in glycosylphosphatidylinositol anchor synthesis of GP49 by Giardia lamblia. Biochim. Biophys. Acta 1483, 69–80. 10.1016/S1388-1981(99)00171-7 10601696

[B62] Sullards M. C. Allegood J. C. Kelly S. Wang E. Haynes C. A. Park H.. (2007). Structure-specific, quantitative methods for analysis of sphingolipids by liquid chromatography-tandem mass spectrometry: inside-out sphingolipidomics. Methods Enzymol. 432, 83–115. 10.1016/S0076-6879(07)32004-1 17954214

[B63] Travers M. A. Sow C. Zirah S. Deregnaucourt C. Chaouch S. Queiroz R. M.. (2016). Deconjugated bile salts produced by extracellular bile-salt hydrolase-like activities from the probiotic *Lactobacillus johnsonii* La1 inhibit *Giardia* duodenalis *in vitro* growth. Front. Microbiol. 7:1453. 10.3389/fmicb.2016.01453 27729900 PMC5037171

[B64] Venable M. E. Lee J. Y. Smyth M. J. Bielawska A. Obeid L. M. (1995). Role of ceramide in cellular senescence. J. Biol. Chem. 270, 30701–30708. 10.1074/jbc.270.51.30701 8530509

[B65] Ye Q. Tian H. Chen B. Shao J. Qin Y. Wen J. (2017). Giardia's primitive GPL biosynthesis pathways with parasitic adaptation ‘patches’: implications for Giardia's evolutionary history and for finding targets against giardiasis. Sci. Rep. 7:9507. 10.1038/s41598-017-10054-1 28842650 PMC5573378

[B66] Yichoy M. Duarte T. T. De Chatterjee A. Mendez T. L. Aguilera K. Y. Roy D.. (2011). Lipid metabolism in Giardia: a post-genomic perspective. Parasitology 138, 267–278. 10.1017/S.0031182010001277 20880419 PMC3132189

[B67] Yuyama K. Sun H. Mitsutake S. Igarashi Y. (2012). Sphingolipid-modulated exosome secretion promotes clearance of amyloid-β by microglia. J Biol Chem. 287, 10977–10989. 10.1074/jbc.M.111.324616 22303002 PMC3322859

[B68] Zhang O. Wilson M. C. Xu W. Hsu F. F. Turk J. Kuhlmann F. M.. (2009). Degradation of host sphingomyelin is essential for Leishmania virulence. PLoS Pathog. 5:e100069. 10.1371/journal.ppat.1000692 20011126 PMC2784226

