# Supplementary figures and images for: A Targeted Mass Spectrometric Analysis Reveals the Presence of a Reduced but Dynamic Sphingolipid Metabolic Pathway in an Ancient Protozoan, Giardia lamblia
Source: Front Cell Infect Microbiol. 2019 Jul 24;9:245. doi: 10.3389/fcimb.2019.00245 (PMC6668603; doi:10.3389/fcimb.2019.00245)

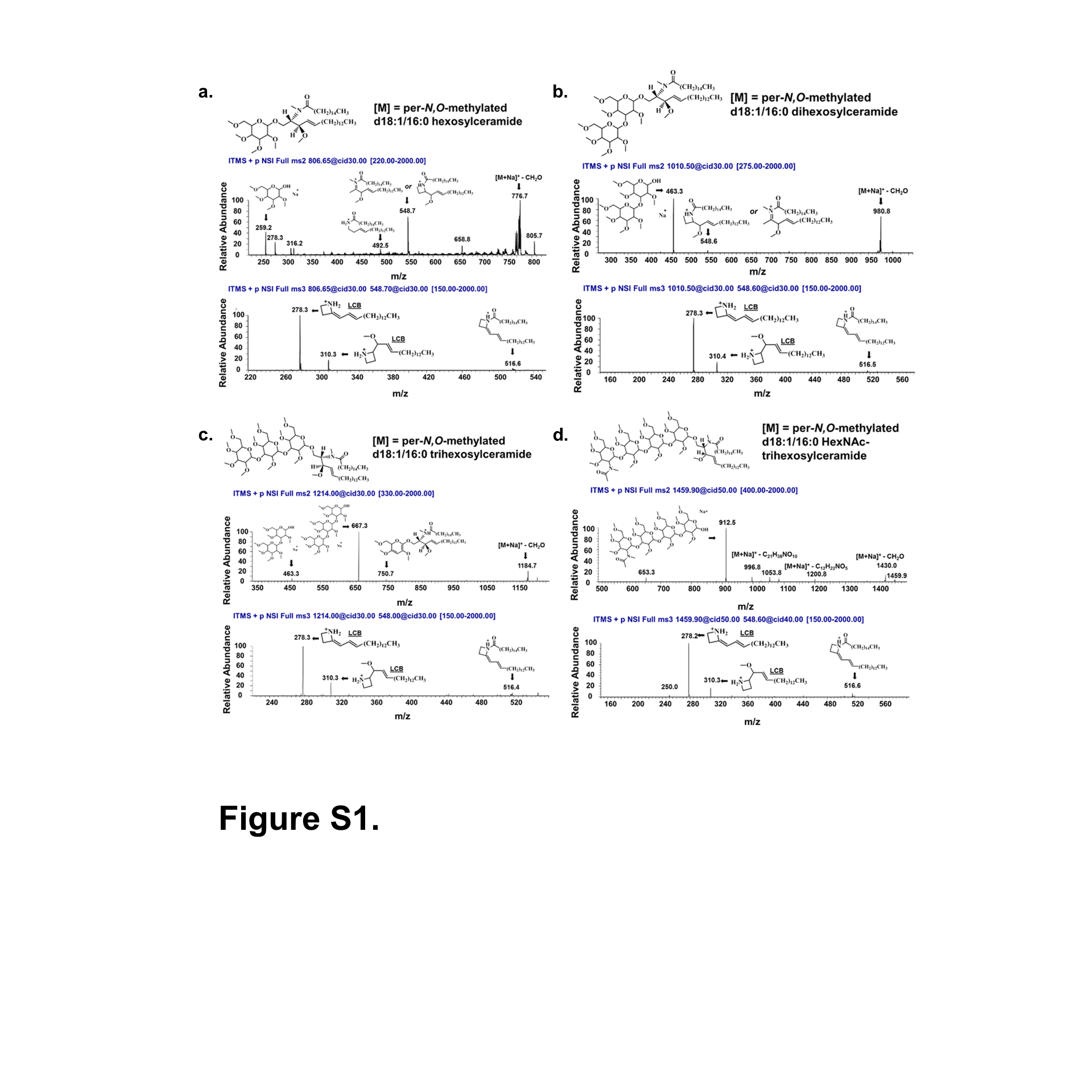

Supplement: Figure S1 — Representative MSn fragmentation analysis with assignment of Cer and LCB. (a) m/z 806.5, Per-N, O-methylated d18:1/16:0-HexCer with Na+ adduct. (b) m/z 1010.5, Per-N, O-methylated d18:1/16:0-Hex2Cer with Na+ adduct. (c) m/z 1214.8, Per-N, O-methylated d18:1/16:0- Hex2Cer with Na+ adduct. (d) m/z 1459.9, Per-N, O-methylated d18:1/16:0-HexNAc-Hex3Cer with Na+ adduct. [file Image_1.TIF]

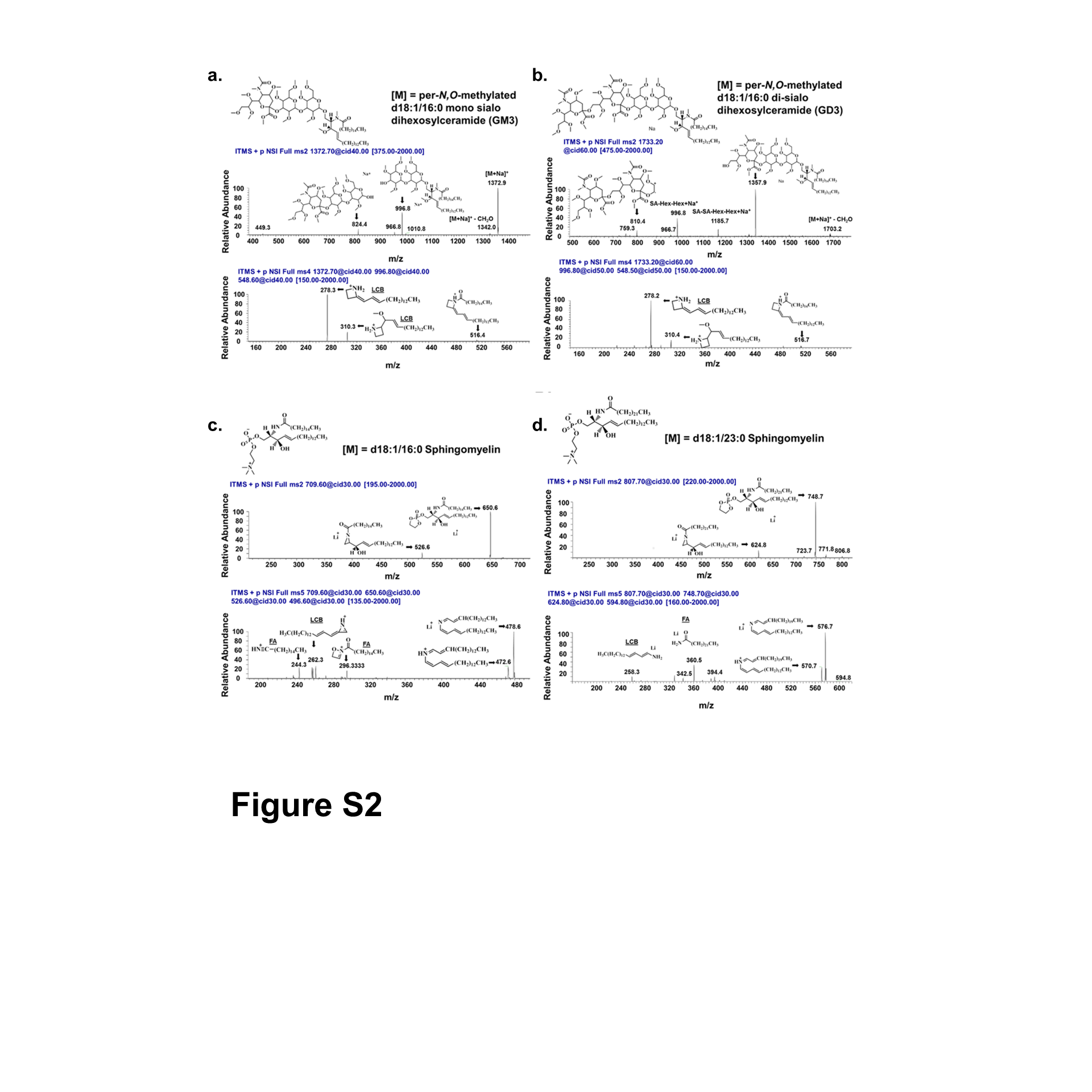

Supplement: Figure S2 — Representative MSn fragmentation analysis with assignment of Cer and LCB. (a) m/z 1372.7, Per-N, O-methylated d18:1/16:0-monosialo-Hex2Cer (GM3) with Na+ adduct. (b) m/z 1733.2, Per-N, O-methylated d18:1/16:0-disialo-Hex2Cer (GM3) with Na+ adduct. (c) m/z 709.6, d18:1/16:0-SM with Li+ adduct. (d) m/z 807.7, d18:1/23:0-SM with Li+ adduct. [file Image_2.TIF]

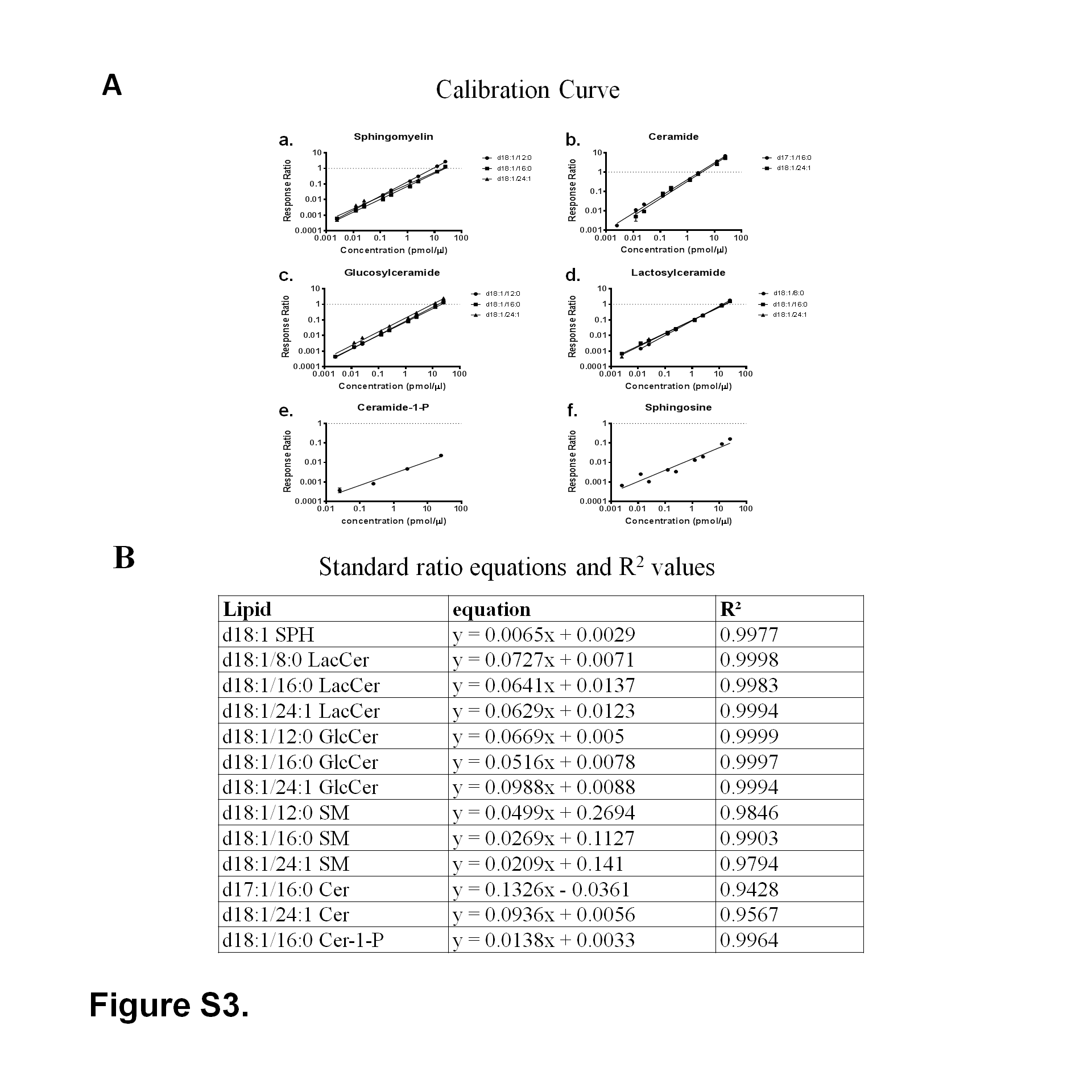

Supplement: Figure S3 — Calibration curves used for quantitation of sphingolipids. For each class, 4–7 concentrations were used to construct the curve. The graph is the plot of the response ratio between varying concentrations of calibration standards and 50 fmol/μl internal standards. For internal standards, both deuterated and non-deuterated (d18:1/12.0 and d18:1/8.0) lipids were used. Equations and R2 values generated were used to transform response ratios to concentration values for each sample. (A) (a) d18:1/12:0-, d18:1/16:0-, and d18:1/24:1-SM; (b) d17:1/16:0- and d18:1/24:1-Cer; (c) d18:1/12:0-, d18:1/16:0-, and d18:1/24:1-glucosylceramide (GlcCer); (d) d18:1/8:0-, d18:1/16:0-, and d18:1/C24:1-lactosylceramide (LacCer); d18:1/16:0-Cer-1-P; and d18:1-Sph. (B) Standard ratio equations and R2 values. [file Image_3.TIF]
