# Supplementary material for: A Targeted Mass Spectrometric Analysis Reveals the Presence of a Reduced but Dynamic Sphingolipid Metabolic Pathway in an Ancient Protozoan, Giardia lamblia
Source: Front Cell Infect Microbiol. 2019 Jul 24;9:245. doi: 10.3389/fcimb.2019.00245 (PMC6668603; doi:10.3389/fcimb.2019.00245)
Supplement: Table S1 — Neutral sphingolipid species identified by direct infusion nano-ESI MS/MS using TIM and MSn fragmentation. [file Table_1.DOC]

**Table S1: Neutral sphingolipid species identified by direct infusion into nano-ESI-MS/MS using TIM and MS^n^ fragmentation.**

| **Sphingolipid species[[1]](#endnote-2)** | **Parent ion *m/z*[[2]](#endnote-3)** | **Adduct ion** | **TIM filter[[3]](#endnote-4)** |
| --- | --- | --- | --- |
| **d18:1/26:0-HexNAc-Hex_3_Cer** | 1590.0 | [Na]^+^ | NL 260 |
| **d18:1/25:0-HexNAc-Hex_3_Cer** | 1586.0 | [Na]^+^ | NL 260 |
| **d18:1/24:0-HexNAc-Hex_3_Cer** | 1572.0 | [Na]^+^ | NL 260 |
| **d18:1/23:0-HexNAc-Hex_3_Cer** | 1558.0 | [Na]^+^ | NL 260 |
| **d18:1/22:0-HexNAc-Hex_3_Cer** | 1544.0 | [Na]^+^ | NL 260 |
| **d18:1/21:0-HexNAc-Hex_3_Cer** | 1530.0 | [Na]^+^ | NL 260 |
| **d18:1/20:0-HexNAc-Hex_3_Cer** | 1516.0 | [Na]^+^ | NL 260 |
| **d18:1/19:0-HexNAc-Hex_3_Cer** | 1502.0 | [Na]^+^ | NL 260 |
| **d18:1/18:0-HexNAc-Hex_3_Cer** | 1488.0 | [Na]^+^ | NL 260 |
| **d18:1/17:0-HexNAc-Hex_3_Cer** | 1474.0 | [Na]^+^ | NL 260 |
| **d18:1/16:0-HexNAc-Hex_3_Cer** | 1460.0 | [Na]^+^ | NL 260 |
| **d18:1/15:0-HexNAc-Hex_3_Cer** | 1446.0 | [Na]^+^ | NL 260 |
| **d18:1/24:0-HexNAc-Hex_4_Cer** | 1734.0 | [Na]^+^ | NL 260 |
| **d18:1/20:0-HexNAc-Hex_4_Cer** | 1706.0 | [Na]^+^ | NL 260 |
| **d18:1/18:0-HexNAc-Hex_4_Cer** | 1678.0 | [Na]^+^ | NL 260 |
| **d18:1/16:0-HexNAc-Hex_4_Cer** | 1650.0 | [Na]^+^ | NL 260 |
| **d18:1/14:0-Hex_3_Cer** | 1186.0 | [Na]^+^ | BP 667 |
| **d18:1/15:0-Hex_3_Cer** | 1200.0 | [Na]^+^ | BP 667 |
| **d18:1/16:0-Hex_3_Cer** | 1214.0 | [Na]^+^ | BP 667 |
| **d18:1/17:0-Hex_3_Cer** | 1228.0 | [Na]^+^ | BP 667 |
| **d18:1/18:0-Hex_3_Cer** | 1242.0 | [Na]^+^ | BP 667 |
| **d18:1/19:0-Hex_3_Cer** | 1256.0 | [Na]^+^ | BP 667 |
| **d18:1/20:0-Hex_3_Cer** | 1270.0 | [Na]^+^ | BP 667 |
| **d18:1/21:0-Hex_3_Cer** | 1284.0 | [Na]^+^ | BP 667 |
| **d18:1/22:0-Hex_3_Cer** | 1298.0 | [Na]^+^ | BP 667 |
| **d18:1/23:0-Hex_3_Cer** | 1312.0 | [Na]^+^ | BP 667 |
| **d18:1/24:0-Hex_3_Cer** | 1326.0 | [Na]^+^ | BP 667 |
| **d18:1/25:0-Hex_3_Cer** | 1340.0 | [Na]^+^ | BP 667 |
| **d18:1/26:0-Hex_3_Cer** | 1354.0 | [Na]^+^ | BP 667 |
| **d18:1/14:0-Hex_2_Cer** | 982.7 | [Na]^+^ | BP 463 |
| **d18:1/15:0-Hex_2_Cer** | 996.7 | [Na]^+^ | BP 463 |
| **d18:1/16:1 Hex_2_Cer** | 1008.6 | [Na]^+^ | BP 463 |
| **d18:1/16:0 Hex_2_Cer** | 1010.7 | [Na]^+^ | BP 463 |
| **d18:1/17:0-Hex_2_Cer** | 1024.8 | [Na]^+^ | BP 463 |
| **d18:1/18:0-Hex_2_Cer** | 1038.8 | [Na]^+^ | BP 463 |
| **d18:1/19:0-Hex_2_Cer** | 1052.8 | [Na]^+^ | BP 463 |
| **d18:1/20:0-Hex_2_Cer** | 1066.8 | [Na]^+^ | BP 463 |
| **d18:1/22:0-Hex_2_Cer** | 1094.8 | [Na]^+^ | BP 463 |
| **d18:1/23:0-Hex_2_Cer** | 1108.9 | [Na]^+^ | BP 463 |
| **d18:1/24:1-Hex_2_Cer** | 1120.9 | [Na]^+^ | BP 463 |
| **d18:1/24:0-Hex_2_Cer** | 1122.9 | [Na]^+^ | BP 463 |
| **d18:1/14:0-HexCer** | 778.6 | [Na]^+^ | BP 259 |
| **d18:1/15:0-HexCer** | 792.6 | [Na]^+^ | BP 259 |
| **d18:1/16:0-HexCer** | 806.6 | [Na]^+^ | BP 259 |
| **d18:1/17:0-HexCer** | 820.7 | [Na]^+^ | BP 259 |
| **d18:1/18:0-HexCer** | 834.7 | [Na]^+^ | BP 259 |
| **d18:1/19:0-HexCer** | 848.6 | [Na]^+^ | BP 259 |
| **d18:1/20:0-HexCer** | 862.6 | [Na]^+^ | BP 259 |
| **d18:1/21:0-HexCer** | 876.6 | [Na]^+^ | BP 259 |
| **d18:1/22:0-HexCer** | 890.6 | [Na]^+^ | BP 259 |
| **d18:1/23:0-HexCer** | 904.6 | [Na]^+^ | BP 259 |
| **d18:1/24:1-HexCer** | 916.8 | [Na]^+^ | BP 259 |
| **d18:1/24:0-HexCer** | 918.8 | [Na]^+^ | BP 259 |
| **d18:1/14:0-Cer** | 574.5 | [Na]^+^ | NL 48 |
| **d18:1/15:0-Cer** | 588.5 | [Na]^+^ | NL 48 |
| **d18:1/16:0-Cer** | 602.5 | [Na]^+^ | NL 48 |
| **d18:1/17:0-Cer** | 616.6 | [Na]^+^ | NL 48 |
| **d18:1/18:0-Cer** | 630.6 | [Na]^+^ | NL 48 |
| **d18:1/19:0-Cer** | 644.6 | [Na]^+^ | NL 48 |
| **d18:1/20:0-Cer** | 658.6 | [Na]^+^ | NL 48 |
| **d18:1/22:0-Cer** | 686.6 | [Na]^+^ | NL 48 |
| **d18:1/23:0-Cer** | 700.7 | [Na]^+^ | NL 48 |
| **d18:1/24:1-Cer** | 712.7 | [Na]^+^ | NL 48 |
| **d18:1/24:0-Cer** | 714.7 | [Na]^+^ | NL 48 |
| **d18:1/15:0-SM** | 695.6[[4]](#endnote-5) | [Li]^+^ | NL 59 |
| **d18:1/16:0-SM** | 709.6 | [Li]^+^ | NL 59 |
| **d18:1/17:0-SM** | 723.6 | [Li]^+^ | NL 59 |
| **d18:1/18:0-SM** | 737.6 | [Li]^+^ | NL 59 |
| **d18:1/19:0-SM** | 751.6 | [Li]^+^ | NL 59 |
| **d18:1/20:0-SM** | 765.6 | [Li]^+^ | NL 59 |
| **d18:1/22:0-SM** | 793.7 | [Li]^+^ | NL 59 |
| **d18:1/23:1-SM** | 805.6 | [Li]^+^ | NL 59 |
| **d18:1/23:0-SM** | 807.7 | [Li]^+^ | NL 59 |
| **d18:1/24:1-SM** | 819.7 | [Li]^+^ | NL 59 |
| **d18:1/24:0-SM** | 821.7 | [Li]^+^ | NL 59 |
| **d18:1/26:0-SM** | 849.7 | [Li]^+^ | NL 59 |

1. HexNAc, *N*-acetyl-hexosamine; Hex, hexose; Cer, ceramide; SM, sphingomyelin [↑](#endnote-ref-2)
2. per-*N,O*-methylated [↑](#endnote-ref-3)
3. NL, neutral loss; BP, base peak [↑](#endnote-ref-4)
4. SM species were not methylated [↑](#endnote-ref-5)
