# Supplementary material for: A Targeted Mass Spectrometric Analysis Reveals the Presence of a Reduced but Dynamic Sphingolipid Metabolic Pathway in an Ancient Protozoan, Giardia lamblia
Source: Front Cell Infect Microbiol. 2019 Jul 24;9:245. doi: 10.3389/fcimb.2019.00245 (PMC6668603; doi:10.3389/fcimb.2019.00245)
Supplement: Table S2 — Acidic sphingolipids identified by direct infusion nano-ESI MS/MS using TIM and MSn fragmentation. [file Table_2.DOC]

**Table S2. Acidic sphingolipid species identified by direct infusion into nano-ESI-MS/MS using TIM and MS^n^ fragmentation.**

| **Sphingolipid species[[1]](#endnote-2)** | **Parent ion *m/z*[[2]](#endnote-3)** | **Adduct ion** | **TIM filter[[3]](#endnote-4)** |
| --- | --- | --- | --- |
| **d18:1/26:0-SA-Hex_2_Cer** | 1512 | [Na]^+^ | NL 376 |
| **d18:1/25:0-SA-Hex_2_Cer** | 1498 | [Na]^+^ | NL 376 |
| **d18:1/24:0-SA-Hex_2_Cer** | 1484 | [Na]^+^ | NL 376 |
| **d18:1/23:0-SA-Hex_2_Cer** | 1470.8 | [Na]^+^ | NL 376 |
| **d18:1/22:0-SA-Hex_2_Cer** | 1456.8 | [Na]^+^ | NL 376 |
| **d18:1/21:0-SA-Hex_2_Cer** | 1442.8 | [Na]^+^ | NL 376 |
| **d18:1/20:0-SA-Hex_2_Cer** | 1428.8 | [Na]^+^ | NL 376 |
| **d18:1/19:0-SA-Hex_2_Cer** | 1414.8 | [Na]^+^ | NL 376 |
| **d18:1/18:0-SA-Hex_2_Cer** | 1400.8 | [Na]^+^ | NL 376 |
| **d18:1/18:1-SA-Hex_2_Cer** | 1398.8 | [Na]^+^ | NL 376 |
| **d18:1/17:0-SA-Hex_2_Cer** | 1386.8 | [Na]^+^ | NL 376 |
| **d18:1/17:1-SA-Hex_2_Cer** | 1384.8 | [Na]^+^ | NL 376 |
| **d18:1/16:0-SA-Hex_2_Cer** | 1372.8 | [Na]^+^ | NL 376 |
| **d18:1/16:1-SA-Hex_2_Cer** | 1370.8 | [Na]^+^ | NL 376 |
| **d18:1/15:0 SA-Hex_2_Cer** | 1358.8 | [Na]^+^ | NL 376 |
| **d18:1/14:0-SA-Hex_2_Cer** | 1344.8 | [Na]^+^ | NL 376 |
| **d18:1/26:0-SA_2_-Hex_2_Cer** | 1874 | [Na]^+^ | NL 376 |
| **d18:1/25:0-SA_2_-Hex_2_Cer** | 1860 | [Na]^+^ | NL 376 |
| **d18:1/24:0-SA_2_-Hex_2_Cer** | 1846 | [Na]^+^ | NL 376 |
| **d18:1/23:0-SA_2_-Hex_2_Cer** | 1832 | [Na]^+^ | NL 376 |
| **d18:1/22:0-SA_2_-Hex_2_Cer** | 1818 | [Na]^+^ | NL 376 |
| **d18:1/21:0-SA_2_-Hex_2_Cer** | 1804 | [Na]^+^ | NL 376 |
| **d18:1/20:0-SA_2_-Hex_2_Cer** | 1790 | [Na]^+^ | NL 376 |
| **d18:1/19:0-SA_2_-Hex_2_Cer** | 1776 | [Na]^+^ | NL 376 |
| **d18:1/18:0-SA_2_-Hex_2_Cer** | 1762.2 | [Na]^+^ | NL 376 |
| **d18:1/17:0-SA_2_-Hex_2_Cer** | 1748.2 | [Na]^+^ | NL 376 |
| **d18:1/16:0-SA_2_-Hex_2_Cer** | 1734.2 | [Na]^+^ | NL 376 |
| **d18:1/15:0-SA_2_-Hex_2_Cer** | 1720.2 | [Na]^+^ | NL 376 |
| **d18:1/16:0-SA-HexNAc-Hex_3_Cer** | 1822 | [Na]^+^ | BP 1273 |
| **d18:1/18:0-SA-HexNAc-Hex_3_Cer** | 1850 | [Na]^+^ | BP 1273 |

1. SA , sialic acid; Hex, hexose; Cer, ceramide; HexNAc, N-acetyl-hexosamine [↑](#endnote-ref-2)
2. Per-*N,O*-methylated [↑](#endnote-ref-3)
3. TIM, total-ion monitoring; NL, neutral loss; BP, base peak [↑](#endnote-ref-4)
