# Supplementary material for: A Targeted Mass Spectrometric Analysis Reveals the Presence of a Reduced but Dynamic Sphingolipid Metabolic Pathway in an Ancient Protozoan, Giardia lamblia
Source: Front Cell Infect Microbiol. 2019 Jul 24;9:245. doi: 10.3389/fcimb.2019.00245 (PMC6668603; doi:10.3389/fcimb.2019.00245)
Supplement: Table S5 — Changes sphingolipid species in non-encysting and encysting trophozoites as well as in water-resistant cysts. Results are shown in percentages and errors (±SD) for each class of sphingolipids. [file Table_5.DOCX]

| Sphingolipid species | Troph  (2.5 x10^6^ cells) | 12 H.P.I.  (2.5 x10^6^ cells) | 24 H.P.I.  (2.5 x10^6^ cells) | Cysts  (2.5 x10^6^ cells) |
| --- | --- | --- | --- | --- |
| **Total d18:0_sphinganine** | **4.85%** Composition 21.64% Deviation | **61.29%** Composition 21.63% Deviation | **28.09%** Composition 18.46% Deviation | **5.77%** Composition 12.37% Deviation |
| **Total d18:1_Sphingosine** | **3.83%** Composition 37.01% Deviation | **66.63%** Composition 21.69% Deviation | **24%**  Composition 19.94% Deviation | **5.54%** Composition 9.59% Deviation |
| **Total ceramide** | **9.86 %** Composition 49.12% Deviation | **39.75%** Composition 19.05% Deviation | **30.77%** Composition 15.39% Deviation | **19.62%** Composition 9.55% Deviation |
| **Cer-1-P d18:1/16:0** | **20.41%** Composition 62.29% Deviation | **46.18%** Composition 19.69% Deviation | **33.41%** Composition 19.36% Deviation | **0%**  Composition N/A Deviation |
| **Total triHexCer** | **11.7%** Composition 45.67% Deviation | **35.47%** Composition 17.83% Deviation | **29.52%** Composition 20.37% Deviation | **23.31%** Composition 9.43% Deviation |
| **Total diHexCer** | **9.43%** Composition 51.65% Deviation | **34.72%** Composition 13.61% Deviation | **29.74%** Composition 12.31% Deviation | **26.1%** Composition 4.60% Deviation |
| **Total HexCer** | **16.23%** Composition 65.82% Deviation | **44.3%** Composition 19.68% Deviation | **28.34%** Composition 14.79% Deviation | **11.13%** Composition 14.41% Deviation |
| **Total sphingomyelin** | **12.8%** Composition  31% Deviation | **34.28%** Composition 14.41% Deviation | **27.77%** Composition  7.94% Deviation | **25.15%** Composition 2.93% Deviation |

**TABLE S5**

Percentage of sphingolipids in various stages of the life cycle of *Giardia*
